# New Thiodiketopiperazine and 3,4-Dihydroisocoumarin Derivatives from the Marine-Derived Fungus *Aspergillus terreus*

**DOI:** 10.3390/md18030132

**Published:** 2020-02-26

**Authors:** Jing-Shuai Wu, Xiao-Hui Shi, Guang-Shan Yao, Chang-Lun Shao, Xiu-Mei Fu, Xiu-Li Zhang, Hua-Shi Guan, Chang-Yun Wang

**Affiliations:** 1 Key Laboratory of Marine Drugs, The Ministry of Education of China, School of Medicine and Pharmacy, Institute of Evolution & Marine Biodiversity, Ocean University of China, Qingdao 266003, China; wujingshuai0110@sina.cn (J.-S.W.); 13061461893@163.com (X.-H.S.); ygshan@126.com (G.-S.Y.); shaochanglun@163.com (C.-L.S.); fuxiumei92@163.com (X.-M.F.); xiulizhang@ouc.edu.cn (X.-L.Z.); 2 Laboratory for Marine Drugs and Bioproducts, Qingdao National Laboratory for Marine Science and Technology, Qingdao 266237, China; 3 Institute of Oceanography, Minjiang University, Fuzhou 350108, China

**Keywords:** marine-derived fungus, *Aspergillus terreus*, thiodiketopiperazines, dihydroisocoumarins

## Abstract

*Aspergillus terreus* has been reported to produce many secondary metabolites that exhibit potential bioactivities, such as antibiotic, hypoglycemic, and lipid-lowering activities. In the present study, two new thiodiketopiperazines, emestrins L (**1**) and M (**2**), together with five known analogues (**3**–**7**), and five known dihydroisocoumarins (**8**–**12**), were obtained from the marine-derived fungus *Aspergillus terreus* RA2905. The structures of the new compounds were elucidated by analysis of the comprehensive spectroscopic data, including high-resolution electrospray ionization mass spectrometry (HRESIMS), one-dimensional (1D) and two-dimensional (2D) nuclear magnetic resonance (NMR), and electronic circular dichroism (ECD) data. This is the first time that the spectroscopic data of compounds **3**, **8**, and **9** have been reported. Compound **3** displayed antibacterial activity against *Pseudomonas aeruginosa* (minimum inhibitory concentration (MIC) = 32 μg/mL) and antifungal activity against *Candida albicans* (MIC = 32 μg/mL). In addition, compound **3** exhibited an inhibitory effect on protein tyrosine phosphatase 1 B (PTP1B), an important hypoglycemic target, with an inhibitory concentration (IC)_50_ value of 12.25 μM.

## 1. Introduction

Marine fungi, and particularly the genus *Aspergillus*, have proven to be a prolific source of structurally novel and biologically active secondary metabolites that play an eminent role in drug discovery progress [1,2]. A wide array of bioactive compounds, including xanthones, alkaloids, cyclic peptides, and terpenes, have been isolated from marine-derived fungi of *Aspergillus* species [3,4]. Among them, thiodiketopiperazines alkaloids (TDKPs) are an important class of secondary metabolites divided into nearly twenty distinct families, and characterized by the presence of a diketopiperazine core featuring thiomethyl groups and/or transannular sulfide bridges [5]. These compounds have been reported to exhibit a broad range of biological properties, including immunosuppressive [6], cytotoxic [7], antibacterial [8], antiviral [9], and anti-angiogenic activities [10]. Specifically, *Aspergillus terreus* has been reported to produce diverse secondary metabolites that display multiple bioactivities, such as antibiotic, hypoglycemic, and lipid-lowering activities [3,4,11].

**Figure 1:**
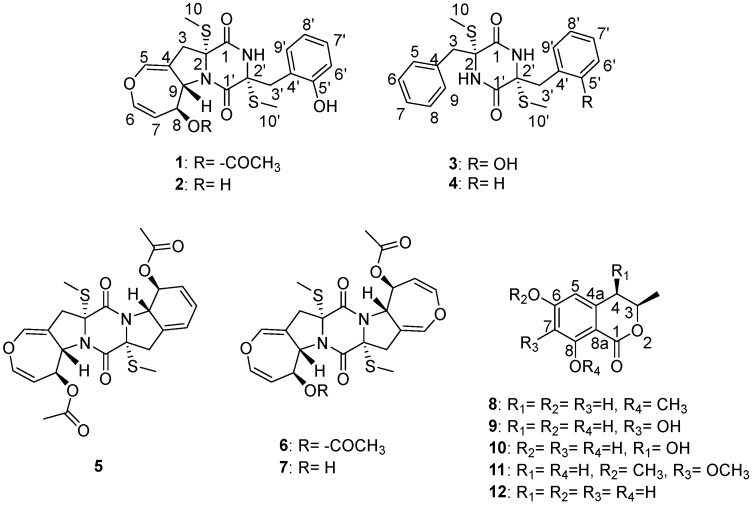
The structures of the compounds.

During our ongoing research for novel bioactive secondary metabolites from marine-derived *Aspergillus* species, we found a series of bioactive natural products with antifungal, antibacterial, antiviral, antifouling, and cytotoxic activities [12]. In the present study, the chemical investigation of the ethyl acetate (EtOAc) extract of *Aspergillus terreus* RA2905, isolated from the fresh inner tissue of the sea hare *Aplysia pulmonica*, resulted in the identification of two new thiodiketopiperazines, emestrins L (**1**) and M (**2**), as well as five known analogues (**3**–**7**), and five known dihydroisocoumarins (**8**–**12**) (Figure 1). Herein, we report the isolation, structural determination, and bioactivity evaluation of these compounds.

## 2. Results

The marine-derived fungus *Aspergillus terreus* RA2905 demonstrated a rapid growth rate on the potato dextrose agar (PDA) plate and produced mature colonies in 3 days. The colonies were characterized by a brown velvety surface (Figure S1). They were cultivated in starch liquid medium at 180 rpm and 28 °C for 7 days. The EtOAc extract (12.5 g) was subjected to column chromatography and semi-preparative high-performance liquid chromatography (HPLC) to yield compounds **1**–**12**, which consisted of two new thiodiketopiperazines, emestrins L (**1**) and M (**2**), five known thiodiketopiperazines, emethacin C (**3**) [13], emethacin B (**4**) [14], bisdethiobis(methylsulfanyl)acetylapoaranotin (**5**) [15], bisdethiobis(methylsulfanyl)acetylaranotin (**6**) [16], and alternarosin A (**7**) [17], and five known dihydroisocoumarins, (3R)-8-methoxy-6-hydroxymellein (**8**) [18], (3R)-6,7,8-trihydroxymellein (**9**) [19], cis-4,6-dihydroxymellein (**10**) [20], (3R)-6,7-dimethoxymellein (**11**) [21], and (3R)-6-hydroxymellein (**12**) [22].

### 2.1. Structure Elucidation

**Figure 2:**
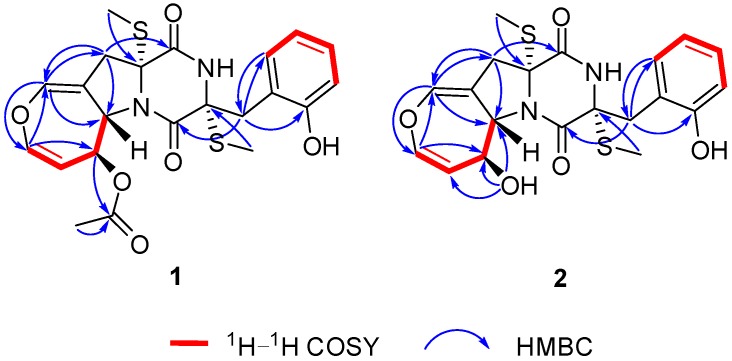
The ^1^H-^1^H Correlation Spectroscopy (COSY) and Key Heteronuclear Multiple Bond Correlation (HMBC) Correlations for **1** and **2**.

Emestrin L (**1**) was obtained as a white powder with the molecular formula C_22_H_24_N_2_O_6_S_2_ established by the HRESIMS spectrum, indicating 12 degrees of unsaturation. The stretch signals at 3600, 3395, 2998, 2913, 1646, 1436, and 1314 cm^‒1^ in the infrared (IR) spectrum suggested the presence of aromatic and carbonyl groups in **1**. The ^1^H NMR spectroscopic data revealed the signal characteristics of the *ortho*-substituted phenyl group (H-6′ to H-9′) (Table 1), which were supported by the corresponding ^1^H-^1^H correlation spectroscopy (COSY) and heteronuclear multiple bond correlation (HMBC) correlations, as shown in Figure 2. A total of 22 carbon atoms, including two thiomethyl carbons (*δ*_H_ 2.13, *δ*_C_ 14.4; *δ*_H_ 2.25, *δ*_C_ 13.8), one acetyl methyl (*δ*_H_ 1.99, *δ*_C_ 21.3), two heteroatom-substituted methane carbons, two methylene carbons, two saturated quaternary carbons (heteroatom-substituted), ten olefinic carbons (seven protonated), and three carbonyl carbons were observed in the ^13^C NMR spectrum (Table 1). The HMBC correlations from H-10 to C-2, from H-10′ to C-2′, from H-3 to C-1, and from H-3′ to C-1′ indicated the presence of a disulfide diketopiperazine skeleton in **1** (Figure 2). The *ortho*-substituted aromatic ring was connected with the diketopiperazine moiety via a methylene bridge of C-3′ based on the HMBC correlations from H-3′ to C-4′/C-5′/C-9′. The ^1^H−^1^H COSY data showed the presence of an isolated spin system corresponding to the C-6−C-7−C-8−C-9 fragment. A 4,5-dihydrooxepine existed in **1** based on the key HMBC correlations from H-5 to C-3/C-6/C-9 and from H-6 to C-5/C-7/C-8, along with the chemical shifts of C-5 (*δ*_C_ 137.6), C-6 (*δ*_C_ 140.3), and C-9 (*δ*_C_ 61.5). The HMBC correlations from H-3 to C-1/C-5/C-9 indicated that the 4,5-dihydrooxepine moiety was located at C-2 of the diketopiperazine core (Figure 2). As the presence of cyclic dipeptide, aromatic ring, 4,5-dihydrooxepine, and the other carbonyl in the molecule occupied 11 degrees of unsaturation, C-9 should be attached to the 2-N atom to form a pyrrolidine ring on the basis of the chemical shifts of C-9 (*δ*_H_ 4.79, *δ*_C_ 61.5). The carbethoxy was anchored at C-8 on the basis of the HMBC correlation from H-8 to C-11. Collectively, these data permitted the assignment of the planar structure of **1**.

**Figure 3:**
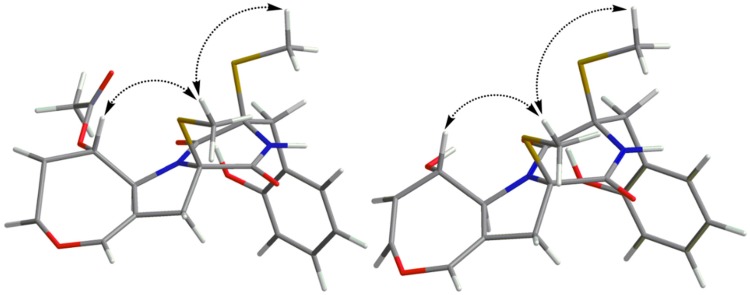
The Nuclear Overhauser Effect (NOE) Correlations for **1** and **2**.

**Figure 4:**
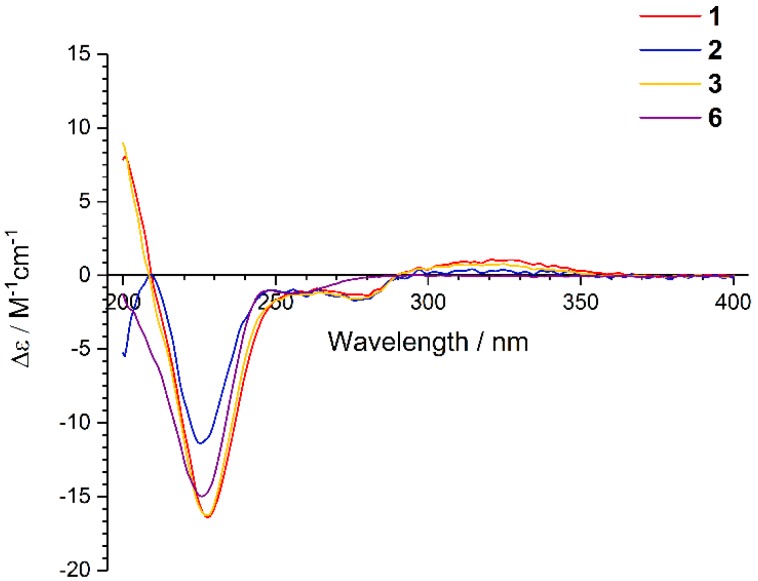
The Electronic Circular Dichroism (ECD) Spectra of **1**–**3** and **6**.

**Figure 5:**
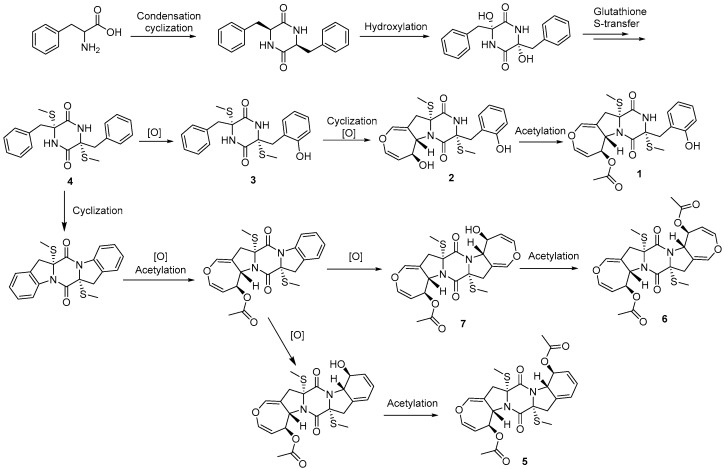
The Possible Biosynthesis Pathway of Thiodiketopiperazines.

The coupling constants of ^3^*J*_H-6–H-7_ = 8.3 Hz indicated that the double bond was a *Z*-configuration. The 7.9 Hz coupling constants between H-8 and H-9 suggested their *anti*-relationship. The nuclear Overhauser effects (NOE) were observed for H-8 and H-10′ when on irradiation of H-10, which demonstrated that they should be on the same face (Figure 3). The absolute configurations of C-2 and C-2′ were assigned as *R* and *R*, respectively, determined by the similar electronic circular dichroism (ECD) data and the same biogenetic pathway as the co-isolated known compound **6** (Figure 4 and Figure 5), for which the absolute configuration was confirmed by X-ray data (Figure S41). Thus, the absolute configurations of **1** were established as 2*R*,2′*R*,8*S*,9*S*.

Emestrin M (**2**) was assigned the molecular formula C_20_H_22_N_2_O_5_S_2_, with 11 degrees of unsaturation, on the basis of its HRESIMS data, less 42 Da compared with **1**. The ^1^H and ^13^C NMR data were very similar to those of **1** (Table 1), which revealed the presence of a thiodiketopiperazine, an *ortho*-substituted phenyl, and a 4,5-dihydrooxepine structure. The main differences were the disappearance of the carbethoxy (*δ*_H_ 1.99, *δ*_C_ 21.3; *δ*_C_ 169.9) in **1**, and the appearance of one hydroxyl (*δ*_H_ 5.44). The hydroxyl was anchored at C-8 based on the HMBC correlations from 8-OH to C-7/C-8/C-9. Thus, the planar structure of **2** was established. The similar coupling constants of **2** and **1** between H-6 and H-7, and between H-8 and H-9, respectively, suggested that the relative configurations of the 4,5-dihydrooxepine of **2** were consistent with those of **1** (Figure 3). The absolute configurations of **2** were identical to those of **1** on the basis of the similar ECD data and of the same biogenetic pathway (Figure 4 and Figure 5).

Emethacin C (**3**) was isolated as a white powder. Its molecular formula was defined as C_20_H_22_N_2_O_3_S_2_ by HRESIMS, with more than 16 Da compared with the known compound emethacin B (**4**). The ^1^H NMR and ^13^C NMR spectroscopic data of **3** were closely related to those of **4**, which revealed the presence of a thiodiketopiperazine structure. The distinction was that one aromatic hydrogen in **4** was replaced by one hydroxyl in **3**. The hydroxyl was anchored at C-5′ based on the ^1^H−^1^H COSY correlations of H-6′/H-7′/H-8′/H-9′ and the HMBC correlations from H-3′ to C-5′/C-9′ (Figure S40). The absolute configurations of **3** were identical to those of **1** and **2** on the basis of the similar ECD data and of the same biogenetic pathway (Figure 4 and Figure 5). It should be noted that the known compound **3** is listed in SciFinder Scholar with the CAS Registry Number 2166398-50-9, but this is the first time that its spectroscopic data have been reported.

(3*R*)-8-methoxy-6-hydroxymellein (**8**) was obtained as a yellow solid with the molecular formula C_11_H_12_O_4_ determined by the HRESIMS data, with more than 14 Da compared with the co-isolated known compound (3*R*)-6-hydroxymellein (**12**). The ^1^H NMR data of **8** displayed the presence of the *meta*-coupled aromatic protons at *δ*_H_ 5.80 (1H, d, *J* = 1.9 Hz) and at *δ*_H_ 5.71 (1H, d, *J* = 1.9 Hz). The ^13^C NMR spectrum revealed 17 carbons, including one carbonyl, six olefinic carbons (two oxygenated), one oxygenated methine carbon, one methylene, and two methyl carbons (one oxygenated). These spectroscopic features were similar to those of (3*R*)-6-hydroxymellein (**12**) except for an additional oxygenated methyl group. The HMBC correlation from H-9 to C-8 was observed for **8** (Figure S42), suggesting that the methoxy group was attached to C-8. The negative Cotton effect at 270 nm in the ECD spectrum of **8** suggested that the absolute configuration of C-3 was *R* (Figure S43) [23]. Compound **8** is listed in SciFinder Scholar with the CAS Registry Number 2247026-31-7, but this is the first time that its spectroscopic data have been reported.

(3*R*)-6,7,8-trihydroxymellein (**9**) was established as C_10_H_10_O_5_ on the basis of the HRESIMS data, with more than 16 Da compared with compound **12**. The ^1^H NMR and ^13^C NMR spectroscopic data of **9** were nearly identical to those of **12,** except that one proton at the aromatic ring in **12** was replaced by one hydroxyl in **9**. The HMBC correlations from H-5 to C-4/C-7/C-8a indicated that the hydroxyl group was located at C-7 (Figure S42). Similar to **8**, the absolute configuration of C-3 in **9** was also determined as *R* by the negative Cotton effect at 270 nm (Figure S44). Compound **9** is also listed in SciFinder Scholar with the CAS Registry Number 2407423-58-7, but this is the first time that its spectroscopic data have been reported.

### 2.2. Bioassays

All of the isolated compounds were tested for their antibacterial, antifungal, cytotoxic, and 1,10-diphenyl-2-picryl-hydazyl (DPPH) scavenging activities. Their protein tyrosine phosphatase 1 B (PTP1B) inhibitory activities were also measured—PTP1B is an important hypoglycemic target in diabetes. We found that compounds **2** and **3** displayed antibacterial activities against *Pseudomonas aeruginosa* ATCC 27853 with minimum inhibitory concentration (MIC) values of 64 μg/mL and 32 μg/mL, respectively. Intriguingly, compound **3** also exhibited antifungal activity against *Candida albicans* ATCC10231 with a MIC value of 32 μg/mL. Compounds **3**, **5**, and **7** showed PTP1B inhibitory activities with inhibitory concentration (IC)_50_ values of 12.25, 25.70 and 24.32 μM, respectively. In addition, compound **9** exhibited a weak DPPH scavenging activity, with an IC_50_ value of 147 μM. All of the isolated compounds showed no cytotoxicity.

## 3. Materials and Methods

### 3.1. Instrumentation

Optical rotations were measured with a JASCO P-1020 digital polarimeter (Jasco Corp., Tokyo, Japan). UV spectra were recorded with a HITACHI UH 5300 UV spectrophotometer (Hitachi, Tokyo, Japan). ECD data were acquired on a J-815-150S Circular Dichroism spectrometer (JASCO Electric Co., Ltd., Tokyo, Japan). IR spectra were recorded with a Nicolet-Nexus-470 spectrometer (Thermo Electron Co., Madison, WI, USA) using KBr pellets. NMR spectra were acquired by a JEOL JEM-ECP NMR spectrometer (500 MHz for ^1^H and 125 MHz for ^13^C, JEOL, Tokyo, Japan), using tetramethylsilane (TMS) as an internal standard. High-resolution electrospray ionization mass spectrometry (HRESIMS) was measured with a Thermo MAT95XP high resolution mass spectrometer (Thermo Fisher Scientific, Bremen, Germany), and electron ionization mass spectrometry (EIMS) spectra with a Thermo DSQ EImass spectrometer (Thermo Fisher Scientific, Bremen, Germany). Single-crystal X-ray crystallographic analysis was performed on an Agilent Xcalibur Eos Gemini diffractometer (Agilent Technologies, Yarnton, England). Samples were analyzed and prepared using a Hitachi L-2000 HPLC system coupled with a Hitachi L-2455 photodiode array detector, and using a semi-preparative C_18_ column (Kromasil 250 mm × 10 mm, 5 μm). Silica gel (Qing Dao Hai Yang Chemical Group Co., Qing Dao China; 300−400 mesh) and Sephadex LH-20 (Amersham Biosciences, Inc., USA) were used for column chromatography (CC). Precoated silica gel plates (Yan Tai Zi Fu Chemical Group Co., Qing Dao China; G60, F-254) were used for thin-layer chromatography. PTP1B (human recombinant) was purchased from Abcam (ab51277).

### 3.2. Fungal Material

The fungal strain *Aspergillus terreus* RA2905 was isolated from a piece of fresh tissue from the inner part of the sea hare *Aplysia pulmonica*, collected from the Weizhou coral reefs in the South China Sea in April 2010. The fungus was identified as *Aspergillus terreus* according to its morphological traits and a molecular protocol by amplification and sequencing of the DNA sequences of the internal transcribed spacer (ITS) region of the ribosomal RNA (rRNA) gene by using ITS 1 and ITS 4. Its GenBank (NCBI) access number is MK611650. The phylogenetic tree of the ITS gene was constructed by the Neighbor-Joining method with the aid of MEGA 7 (Figure S1). The strain was deposited in the Key Laboratory of Marine Drugs, Ministry of Education of China, School of Medicine and Pharmacy, Ocean University of China, Qingdao, P. R. China.

### 3.3. Extraction and Isolation

Sixty 500-mL Erlenmeyer flasks of the fungal strain were cultivated in a starch liquid medium (soluble starch 10 g/L, peptone 1 g/L, artificial sea salt 30 g/L, 200 mL each flask) at 150 rpm and 28 °C for 7 days. The fermentation broth was filtered through a cheesecloth and extracted repeatedly with an equal amount of EtOAc three times, and then it was evaporated in vacuo to obtain an EtOAc extract (12.5 g). The crude extract was isolated on silica gel CC using a step gradient elution with petroleum ether/EtOAc (10:1 to 1:4, v/v) to provide five fractions (Fr.1−Fr.5). Fr.3 was subjected to a silica gel CC eluted with Hexane/CH_2_Cl_2_/MeOH (10:1:0 to 0:1:1) to obtain four subfractions (Fr.3.1−Fr.3.4). Fr.3.2 was further separated by the semi-preparative HPLC with MeOH/H_2_O (60:40) to give compounds **8** (5.5 mg) and **12** (7.1 mg). Fr.3.3 was separated by Sephadex LH-20 CC eluted with CH_2_Cl_2_/MeOH (1:1) and the semi-preparative HPLC with MeOH/H_2_O (45:55) further, to obtain compounds **9**−**11** (3.2 mg, 3.7 mg, and 2.1 mg, respectively). Fr.4 was separated on a silica gel CC eluted with CH_2_Cl_2_/MeOH (100:1 to 1:1) to provide four subfractions (Fr.4.1−Fr.4.4). Fr.4.1 was further subjected to Sephadex LH-20 CC eluted with CH_2_Cl_2_/MeOH (1:1) to obtain compounds **6** and **7**. Fr.4.3 was separated by the semi-preparative HPLC with MeOH/H_2_O (45:55) to give compounds **1**−**5** (3.3 mg, 3.1 mg, 4.2 mg, 4.5 mg, and 4.3 mg, respectively).

*Emestrin L* (**1**). White powder; [*α*]${}_{D}^{20}$-70.4 (*c* 0.42, MeOH); UV (MeOH) λ_max_ (log *ε*) 229 (3.83), 275 (1.61) nm; IR (KBr) *v*_max_ 3600, 3395, 2998, 2913, 1646, 1436, 1314 cm^−1^; ^1^H and ^13^C NMR (see Table 1); (–)-HRESIMS *m*/*z* 475.1007 [M − H]^−^ (calcd for C_22_H_23_N_2_O_6_S_2_, 475.1003).

*Emestrin M* (**2**). White powder; [*α*]${}_{D}^{20}$-85.1 (*c* 0.43, MeOH); UV (MeOH) λ_max_ (log *ε*) 229 (3.75), 275 (1.75) nm; IR (KBr) *v*_max_ 3600, 3000, 2912, 2138, 1663, 1438, 1315, 1043 cm^−1^; ^1^H NMR (500 MHz, DMSO-*d*_6_) and ^13^C NMR (125 MHz, DMSO-*d*_6_) (see Table 1); (–)-HRESIMS *m*/*z* 433.0896 [M − H]^−^ (calcd for C_20_H_21_N_2_O_5_S_2_, 433.0897).

*Emethacin C* (**3**). White powder; [*α*]${}_{D}^{20}$-64.4 (*c* 0.52, MeOH); UV (MeOH) λ_max_ (log *ε*) 229 (3.89), 275 (2.54) nm; IR (KBr) *v*_max_ 2952, 2868, 1802, 1715, 1459, 1200, 1139 cm^−1^; ^1^H NMR (500 MHz, DMSO-*d*_6_): 8.97 (1H, s, 2-NH), 8.36 (1H, br s, 2′-NH), 7.18 (1H, m, H-7), 7.10 (4H, m, H-5, H-6, H-8, H-9), 6.89 (1H, td, *J* = 7.8, 1.7 Hz, H-7′), 6.67 (1H, td, *J* = 7.8, 1.2 Hz, H-8′), 6.26 (1H, dd, *J* = 7.8, 1.7 Hz, H-9′), 6.22 (1H, dd, *J* =7.8, 1.2 Hz, H-6′), 3.47 (1H, d, *J* = 13.6 Hz, H-3a), 3.05 (1H, d, *J* = 15.4 Hz, H-3′a), 3.01 (1H, d, *J* = 15.4 Hz, H-3′b), 2.96 (1H, d, *J* = 13.6 Hz, H-3b), 2.25 (3H, s, H-10), 2.17 (3H, s, H-10′); ^13^C NMR (125 MHz, DMSO-*d*_6_): 166.3 (C, C-1′), 165.6 (C, C-1), 155.6 (C, C-5′), 135.4 (C, C-4), 130.8 (CH × 2, C-5, C-9), 129.9 (CH, C-9′), 128.3(CH×2, C-6, C-8), 127.7 (CH, C-7′), 127.2 (CH, C-7), 122.3 (C, C-4′), 119.4 (CH, C-6′), 115.2 (CH, C-8′), 65.5 (C, C-2), 65.2 (C, C-2′), 43.5 (CH_2_, C-3), 37.0 (CH_2_, C-3′), 14.0 (CH_3_, C-10), 13.9 (CH_3_, C-10′); (–)-HRESIMS *m*/*z* 401.1003 [M − H]^−^ (calcd for C_20_H_21_N_2_O_3_S_2_, 401.0999).

*(3R)-8-methoxy-6-hydroxymellein* (**8**). Yellow solid; [*α*]${}_{D}^{20}$-47.8 (*c* 1.00, MeOH); UV (MeOH) *λ*_max_ (log *ε*) 238 (2.11), 251 (0.95), 319 (0.41) nm; IR (KBr) *ν*_max_ 3749, 3674, 2360, 1736, 1581, 1418 cm^−1^; ^1^H NMR (500 MHz, DMSO-*d*_6_): 5.80 (1H, d, *J* = 1.9 Hz, H-5), 5.71 (1H, d, *J* = 1.9 Hz, H-7), 4.25 (1H, m, H-3), 3.59 (3H, s, H-9), 2.57 (1H, dd, *J* = 15.8, 3.3 Hz, H-4a), 2.55 (1H, m, overlapped, H-4b), 1.26 (3H, d, *J* = 6.2 Hz, H-10); ^13^C NMR (125 MHz, DMSO-*d*_6_): 172.3 (C, C-1), 163.4 (C-6), 162.1 (C, C-8), 142.5 (C, C-4a), 109.4 (CH, C-5), 100.1 (C, C-8a), 97.1 (CH, C-7), 72.0 (CH, C-3), 54.7 (CH_3_, C-9), 36.2 (CH_2_, C-4), 20.6 (CH_3_, C-10); (+)-HRESIMS *m*/*z* 209.0809 [M + H]^+^ (calcd for C_11_H_13_O_4_, 209.0808).

*(3R)-6,7,8-trihydroxymellein* (**9**). Yellow solid; [*α*]${}_{D}^{20}$-50.1 (*c* 0.37, MeOH); UV (MeOH) *λ*_max_ (log *ε*) 238 (2.03), 251 (1.05), 317 (0.23) nm; IR (KBr) *ν*_max_ 3749, 3674, 2360, 1736, 1651, 1384 cm^−1^; ^1^H NMR (500 MHz, DMSO-*d*_6_): 6.26 (1H, s, H-5), 4.64 (1H, m, H-3), 2.82 (1H, dd, *J* = 16.2, 3.3 Hz, H-4a), 2.71 (1H, dd, *J* = 16.2, 1.3 Hz, H-4b), 1.37 (3H, d, *J* = 6.2 Hz, H-9); ^13^C NMR (125 MHz, DMSO-*d*_6_): 170.0 (C, C-1), 153.2 (C-6), 151.1 (C, C-8), 131.3 (C, C-7), 131.0 (C, C-4a), 106.5 (CH, C-5), 100.0 (C, C-8a), 75.9 (CH, C-3), 33.2 (CH_2_, C-4), 20.4 (CH_3_, C-9); (–)-HRESIMS *m*/*z* 209.0458 [M − H]^−^ (calcd for C_10_H_9_O_5_, 209.0455).

*Crystal data for***6**: C_18_H_18_N_2_O_7_S_2_, *M*r = 534.11, monoclinic, *a* = 6.8830(3) Å, *b* = 12.4383(5) Å, *c* = 14.6020(6) Å, *α* = 90.00°, *β* = 94.415(4)°, *γ* = 90.00°, *V* = 1246.41(9) Å^3^, space group *P*21, *Z* = 2, *D*x = 1.135 mg/m^3^, *μ* (Cu Kα) = 0.670 mm^–1^, and *F* (000) = 425. Crystal dimensions: 0.12 × 0.11 × 0.11 mm^3^. Independent reflections: 7369/4117 (*R*_int_ = 0.0422). The final *R*1 value was 0.0481, *wR*2 = 0.1180 (*I* > 2*σ*(*I*)). Flack parameter = –0.012(13). Crystallographic data for **6** are deposited at the Cambridge Crystallographic Data Centre as supplementary publication number CCDC 1911625.

### 3.4. Antibacterial Assays

The antibacterial activity was evaluated following the standards recommended by the Clinical and Laboratory Standards Institute [24]. Six pathogenic bacterial strains, *Staphylococcus epidermidis* ATCC 12228, *Staphylococcus aureus* ATCC 25923, *Pseudomonas aeruginosa* ATCC 27853, *Bacillus cereus* ATCC 14579, *Escherichia coli* ATCC 25922, and *Sarcina lutea* ATCC 9341, were used, and vancomycin was used as a positive control.

### 3.5. Antifungal Assays

The antifungal bioassays were conducted following the standards recommended by the Clinical and Laboratory Standards Institute [24]. Three pathogenic fungal strains, *Candida albicans* ATCC 24433, *Candida tropicalis* ATCC 20962, and *Candida parapsilosis* ATCC 22019, were used. Amphotericin B was used as a positive control.

### 3.6. PTP1B Inhibition Assays

The PTP1B Inhibition assay was performed in 96-well plates [25]. The compound (10 μL) was added to the 99-μL reaction buffer solution, which consisted of 10 mM Tris (pH 7.4), 50 mM NaCl, 2 mM dithiothreitol (DTT), 1 mM MnCl_2_, and 10 mM para-nitrophenyl phosphate (*p*NPP). The reaction mix was pre-warmed using a block heater at 37 °C. The recombinant PTP1B solution (1 mg/mL, 1 μL) was mixed in each well. An NaOH solution (10 μL, 0.1 M) was added to stop the reaction. The absorbance was recorded at 405 nm using a microplate. Sodium vanadate was used as a positive control.

### 3.7. DPPH Scavenging Activities

The DPPH scavenging assays were performed using the method described by Aquino et al. [26]. The reaction mixture consisted of freshly prepared 100 μM DPPH in methanol, mixed with different concentrations of the compounds. The reaction mixture was incubated for 20 min at room temperature in the dark, and the optical density was recorded at 517 nm.

### 3.8. Cytotoxicity Assays

The cytotoxic activities were evaluated with the sulforhodamine B (SRB) assay [27], using five human tumor cell lines: A549, HCT116, MCF-7, Hela, and Hep G2. Adriamycin was used as a positive control.

## Figures and Tables

**Table 1:** ^1^H Nuclear Magnetic Resonance (NMR) and ^13^C NMR Data for **1** and **2** ^a^.

Position	1		2	
	*δ*_C_, Type	*δ*_H_ (*J* in Hz)	*δ*_C_, Type	*δ*_H_ (*J* in Hz)
1	165.0, C		166.6, C	
2	70.1, C		68.9, C	
3	39.9, CH_2_	2.78, d (15.0)	39.9, CH_2_	2.74, d (15.0)
		1.98, d (15.0)		1.92, d (15.0)
4	110.4, C		109.5, C	
5	137.6, CH	6.70, t (2.3)	137.2, CH	6.60, t (2.3)
6	140.3, CH	6.44, dd (8.3, 2.3)	138.2, CH	6.28, dd (8.3, 2.3)
7	105.9, CH	4.71, dd (8.3, 2.0)	111.4, CH	4.81, dd (8.3, 2.0)
8	71.5, CH	5.67, dt (7.9, 2.0)	71.8, CH	4.43, dt (7.9, 2.0)
9	61.5, CH	4.79, d (7.9)	64.9, CH	4.54, d (7.9)
10	14.4, CH_3_	2.13, s	14.4, CH_3_	2.17, s
11	169.9, C			
12	21.3, CH_3_	1.99, s		
1′	164.8, C		165.0, C	
2′	68.2, C		68.4, C	
3′	39.1, CH_2_	3.43, d (13.7)	39.2, CH_2_	3.45, d (13.7)
		3.19, d (13.7)		3.19, d (13.7)
4′	121.7, C		121.4, C	
5′	156.3, C		156.2, C	
6′	115.8, CH	6.79, dd (8.0, 1.3)	115.7, CH	6.79, dd (8.0, 1.3)
7′	128.8, CH	7.03, td (8.0, 1.7)	128.8, CH	7.04, td (8.0, 1.7)
8′	119.3, CH	6.67, td (7.7, 1.3)	119.2, CH	6.68, dd (7.8, 1.3)
9′	131.7, CH	6.92, dd (7.7, 1.7)	131.8, CH	6.95, dd (7.8, 1.7)
10′	13.8, CH_3_	2.25, s	14.0, CH_3_	2.29, s
8-OH				5.44, d (6.0)

^a^ 500 MHz for ^1^H NMR and 125 MHz for ^13^C NMR in Dimethyl Sulfoxide (DMSO)-*d*_6_.

## Supplementary Materials

The following are available online at https://www.mdpi.com/1660-3397/18/3/132/s1, Figure S1. *Aspergillus terreus* RA2905 and its phylogenetic tree of ITS gene, Figures S2−S39: the NMR, ESIMS and HRESIMS spectra, Figure S40: Key HMBC correlations of compound **3**, Figure S41: crystal diagram of compound **6**, Figure S42: key HMBC correlations of compounds **8** and **9**, Figure S43: the CD spectrum of compound **8**, Figure S44: the CD spectrum of compound **9**.
