# Supplementary material for: New Thiodiketopiperazine and 3,4-Dihydroisocoumarin Derivatives from the Marine-Derived Fungus Aspergillus terreus
Source: Mar Drugs. 2020 Feb 26;18(3):132. doi: 10.3390/md18030132 (PMC7143538; doi:10.3390/md18030132)
Supplement: Supplementary file 1 [file marinedrugs-18-00132-s001.pdf]

Supplementary Information

New Thiodiketopiperazine and 3,4-Dihydroisocoumarin Derivatives from the Marine-derived Fungus *Aspergillus terreus*

Jing-Shuai Wu[1,2], Xiao-Hui Shi[1,2], Guang-Shan Yao[1,2,3], Chang-Lun Shao[1,2], Xiu-Mei Fu[1,2], Xiu-Li Zhang[1,2], Hua-Shi Guan [1,2,*] and Chang-Yun Wang[1,2,*]

1 Key Laboratory of Marine Drugs, The Ministry of Education of China, School of Medicine and Pharmacy; Institute of Evolution & Marine Biodiversity, Ocean University of China, Qingdao 266003, China; wujingshuai0110@sina.cn (J.-S.W.); 13061461893@163.com (X.-H.S.); ygshan@126.com (G.-S.Y.); shaochanglun@163.com (C.-L.S.); fuxiumei92@163.com (X.-M.F.); xiulizhang@ouc.edu.cn (X.-L.Z.)

2 Laboratory for Marine Drugs and Bioproducts, Qingdao National Laboratory for Marine Science and Technology, Qingdao 266237, China

3 Institute of Oceanography, Minjiang University, Fuzhou 350108, China

* Correspondence: hsguan@ouc.edu.cn (H.-S.G.); changyun@ouc.edu.cn (C.-Y.W.); Tel.: +86-53282031667 (H.-S.G.); +86-53282031536 (C.-Y.W.)

List of supporting information

Figure S1. *Aspergillus terreus* RA2905 and its phylogenetic tree of ITS gene

Figure S2. ^{1}H NMR (500 MHz, DMSO-d_6) spectrum of compound **1**

Figure S3. Partial ^{1}H NMR (500 MHz, DMSO-d_6) spectrum of compound **1**

Figure S4. ^{13}C NMR (125 MHz, DMSO-d_6) spectrum of compound **1**

Figure S5. HSQC (DMSO-d_6) spectrum of compound **1**

Figure S6. ^{1}H–^{1}H COSY (DMSO-d_6) spectrum of compound **1**

Figure S7. HMBC (DMSO-d_6) spectrum of compound **1**

Figure S8. NOE (DMSO-d_6) spectrum of compound **1**

Figure S9. HRESIMS spectrum of compound **1**

Figure S10. ESIMS spectrum of compound **1**

Figure S11. ^{1}H NMR (500 MHz, DMSO-d_6) spectrum of compound **2**

Figure S12. Partial ^{1}H NMR (500 MHz, DMSO-d_6) spectrum of compound **2**

Figure S13. ^{13}C NMR (125 MHz, DMSO-d_6) spectrum of compound **2**

Figure S14. HSQC (DMSO-d_6) spectrum of compound **2**

Figure S15. ^{1}H–^{1}H COSY (DMSO-d_6) spectrum of compound **2**

Figure S16. HMBC (DMSO-d_6) spectrum of compound **2**

Figure S17. NOE (DMSO-d_6) spectrum of compound **2**

Figure S18. HRESIMS spectrum of compound **2**

Figure S19. ESIMS spectrum of compound **2**

Figure S20. ^{1}H NMR (500 MHz, DMSO-d_6) spectrum of compound **3**

Figure S21. Partial ^{1}H NMR (500 MHz, DMSO-d_6) spectrum of compound **3**

Figure S22. ^{13}C NMR (125 MHz, DMSO-d_6) spectrum of compound **3**

Figure S23. HSQC (DMSO-d_6) spectrum of compound **3**

Figure S24. ^{1}H–^{1}H COSY (DMSO-d_6) spectrum of compound **3**

Figure S25. HMBC (DMSO-d_6) spectrum of compound **3**

Figure S26. HRESIMS spectrum of compound **3**

Figure S27. ESIMS spectrum of compound **3**

Figure S28. ^{1}H NMR (500 MHz, DMSO-d_6) spectrum of compound **8**

Figure S29. ^{13}C NMR (125 MHz, DMSO-d_6) spectrum of compound **8**

Figure S30. HSQC (DMSO-d_6) spectrum of compound **8**

Figure S31. ^{1}H–^{1}H COSY (DMSO-d_6) spectrum of compound **8**

Figure S32. HMBC (DMSO-d_6) spectrum of compound **8**

Figure S33. HRESIMS spectrum of compound **8**

Figure S34. ^{1}H NMR (500 MHz, DMSO-d_6) spectrum of compound **9**

Figure S35. ^{13}C NMR (125 MHz, DMSO-d_6) spectrum of compound **9**

Figure S36. HSQC (DMSO-d_6) spectrum of compound **9**

Figure S37. ^{1}H–^{1}H COSY (DMSO-d_6) spectrum of compound **9**

Figure S38. HMBC (DMSO-d_6) spectrum of compound **9**

Figure S39. HRESIMS spectrum of compound **9**

Figure S40. Key HMBC correlations of compound **3**

Figure S41. Crystal diagram of compound **6**

Figure S42. Key HMBC correlations of compounds **8** and **9**

Figure S43. The CD spectrum of compound **8**

Figure S44. The CD spectrum of compound **9**

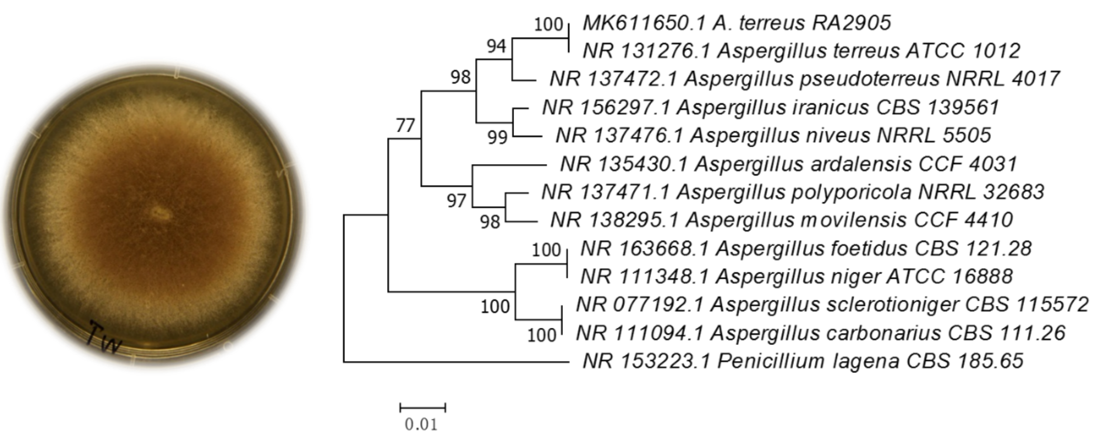

Figure S1. *Aspergillus terreus* RA2905 and its phylogenetic tree of ITS gene

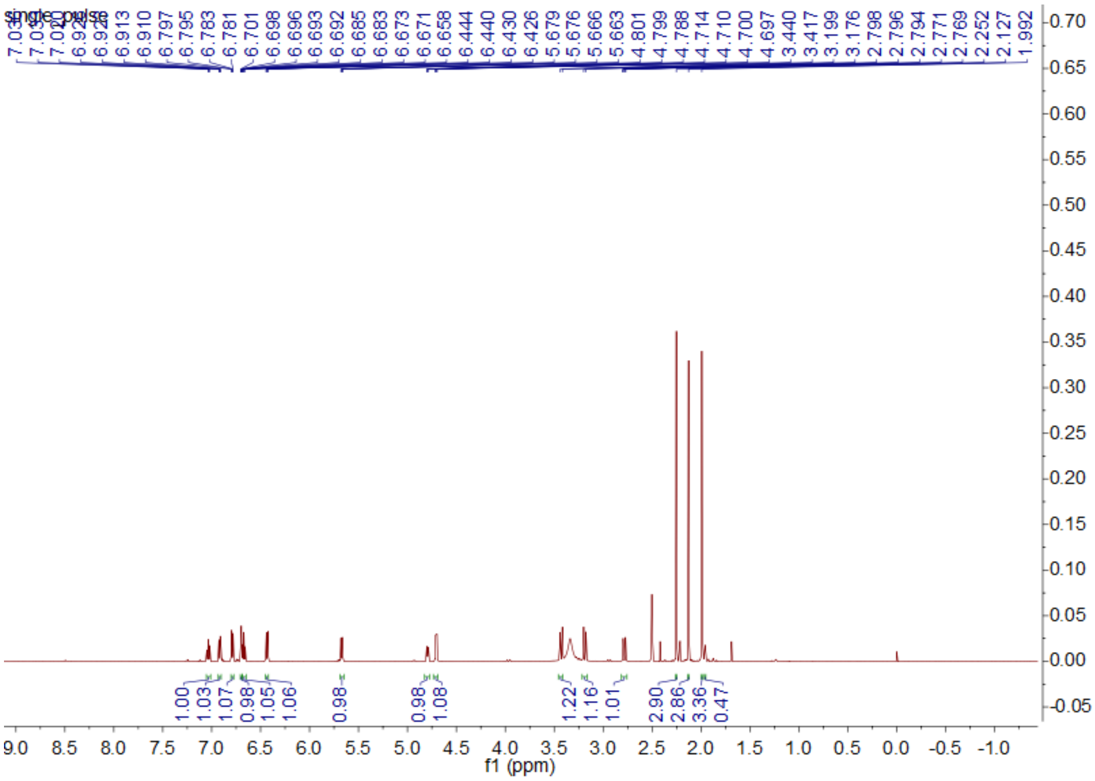

Figure S2. ^{1}H NMR (500 MHz, DMSO-d_6) spectrum of compound **1**

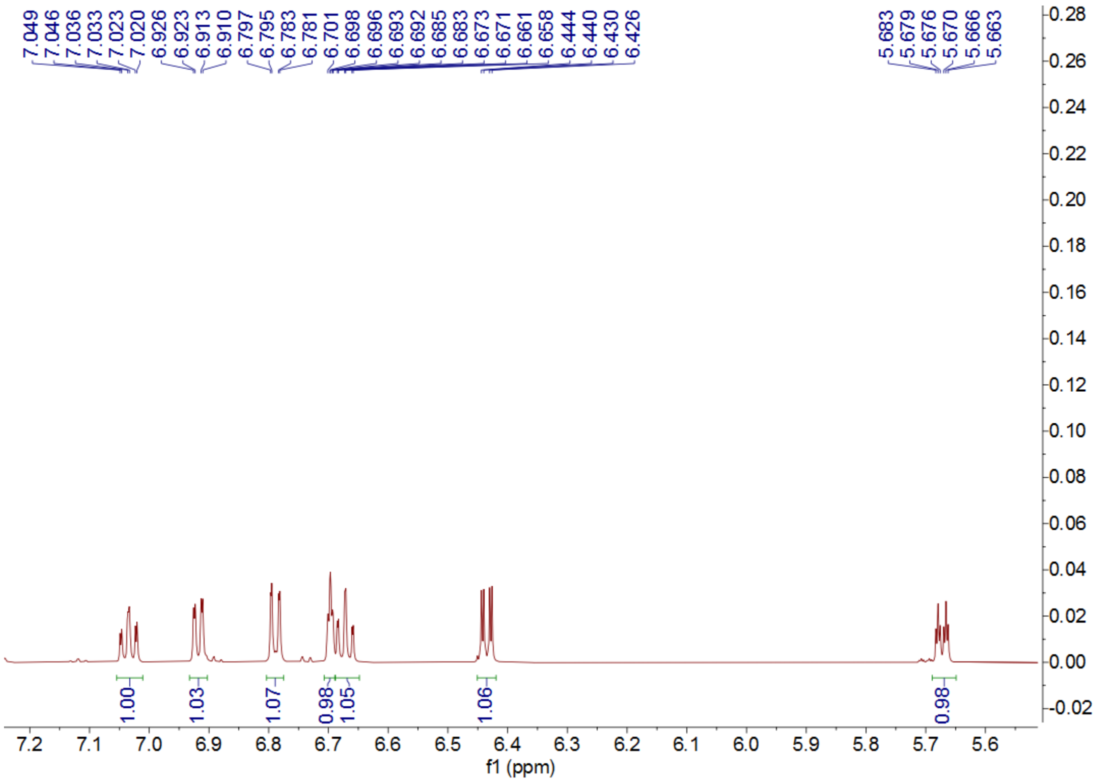

Figure S3. Partial ^{1}H NMR (500 MHz, DMSO-d_6) spectrum of compound **1**

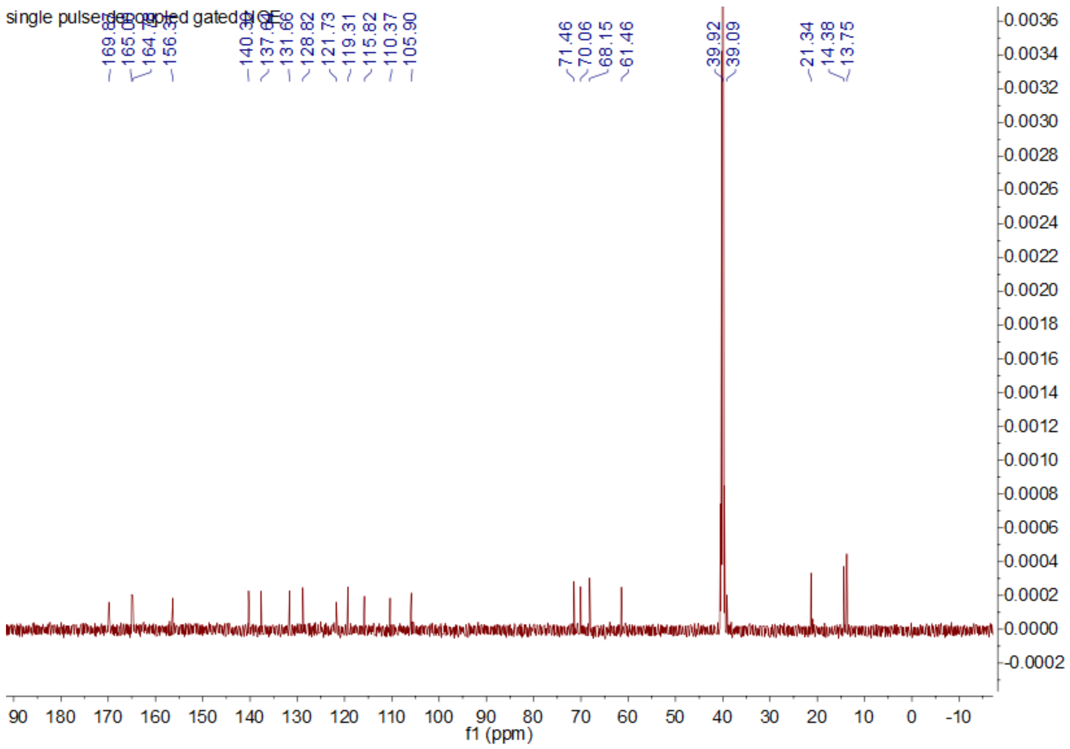

Figure S4. ^{13}C NMR (125 MHz, DMSO-d_6) spectrum of compound **1**

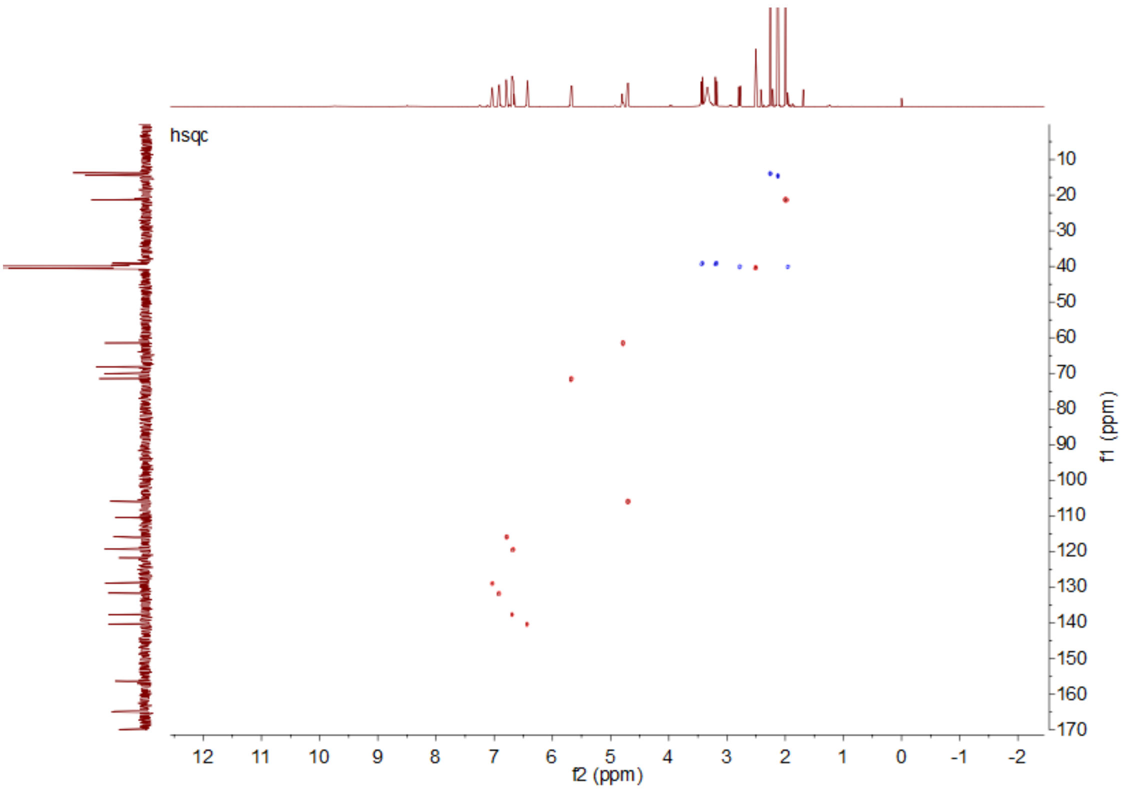

Figure S5. HSQC (DMSO-d_6) spectrum of compound **1**

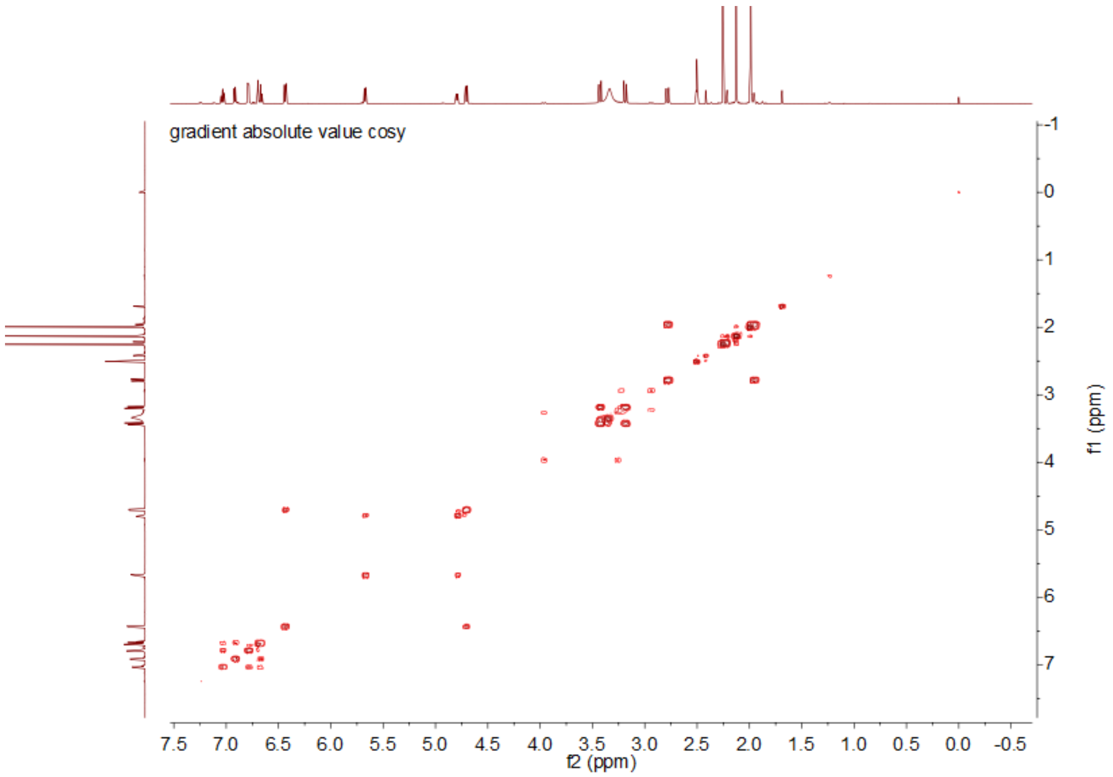

Figure S6. ^{1}H–^{1}H COSY (DMSO-d_6) spectrum of compound **1**

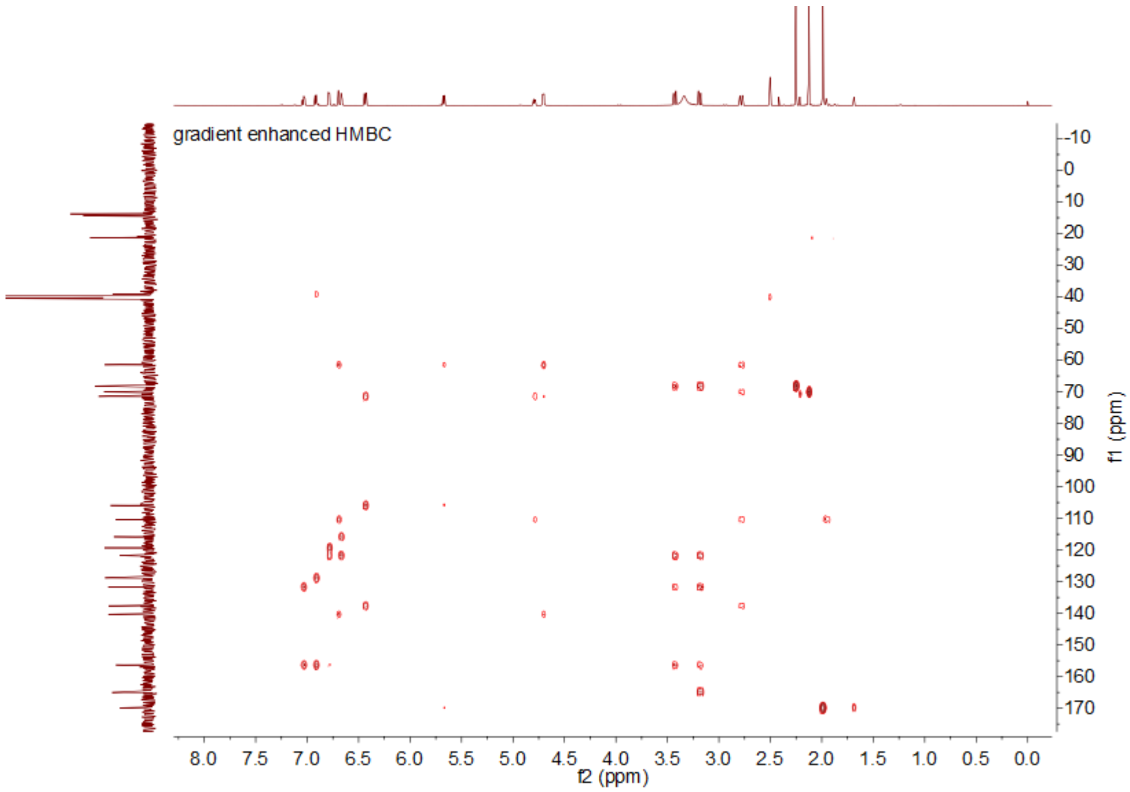

Figure S7. HMBC (DMSO-d_6) spectrum of compound **1**

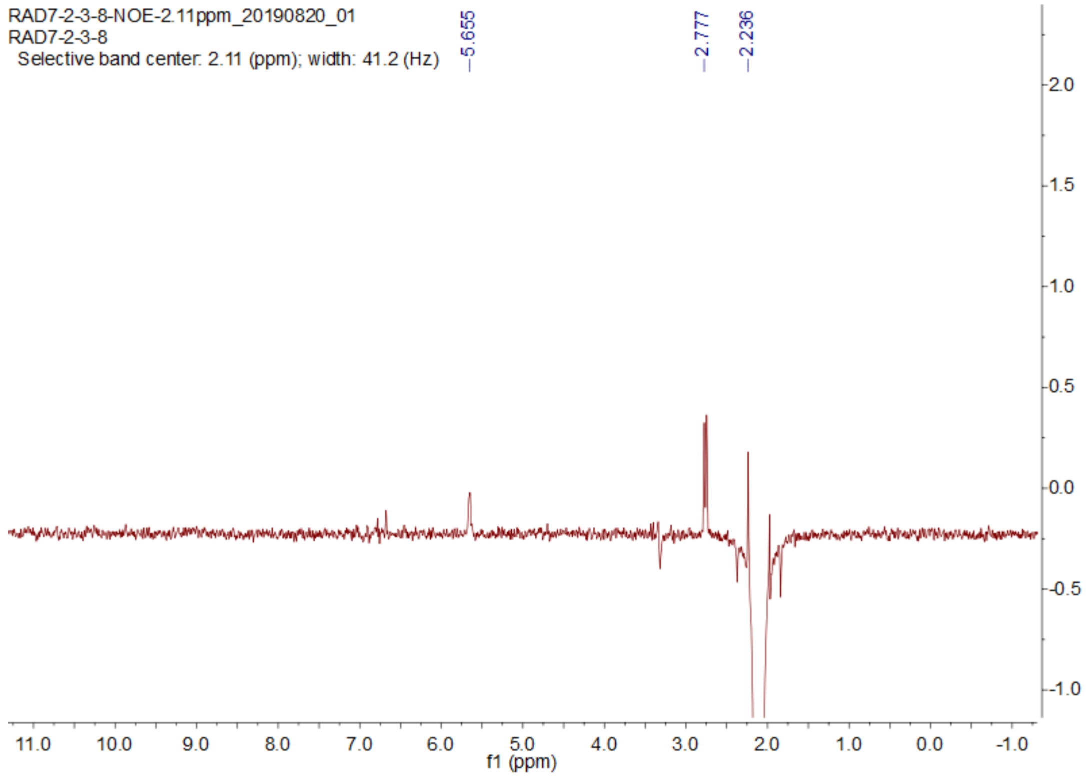

Figure S8. NOE (DMSO-d_6) spectrum of compound **1**

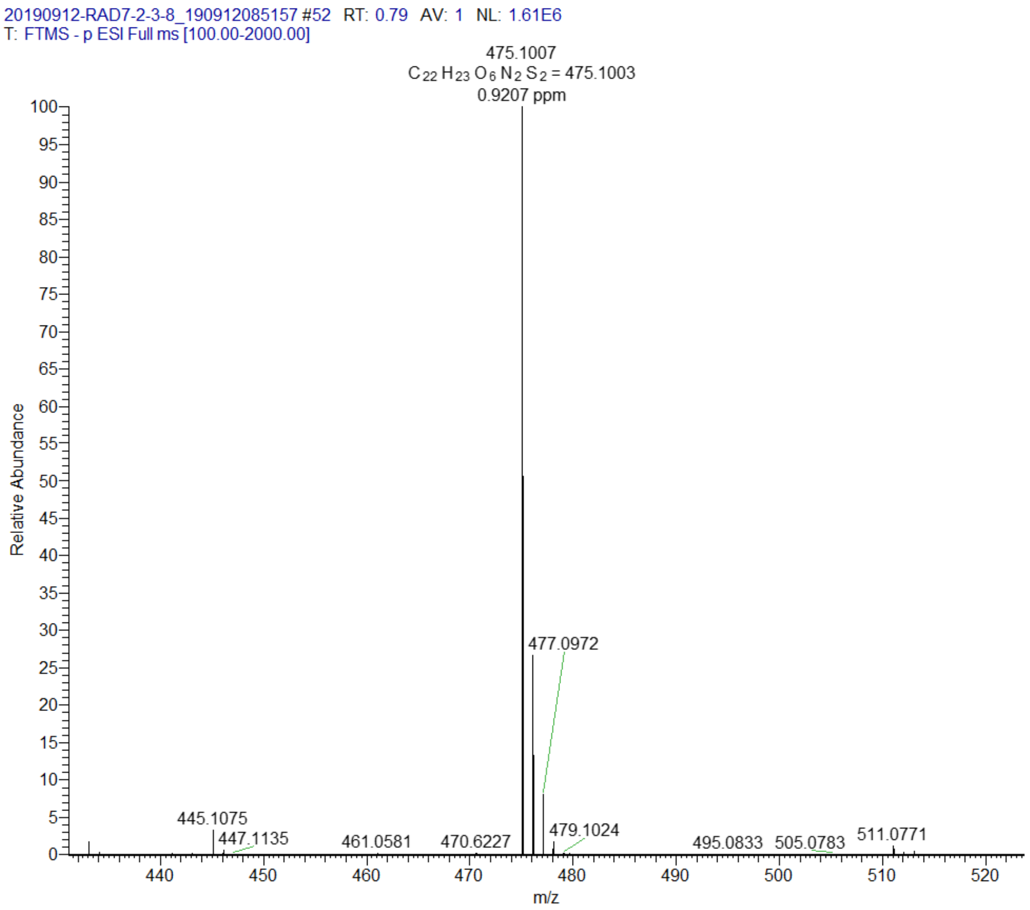

Figure S9. HRESIMS spectrum of compound **1**

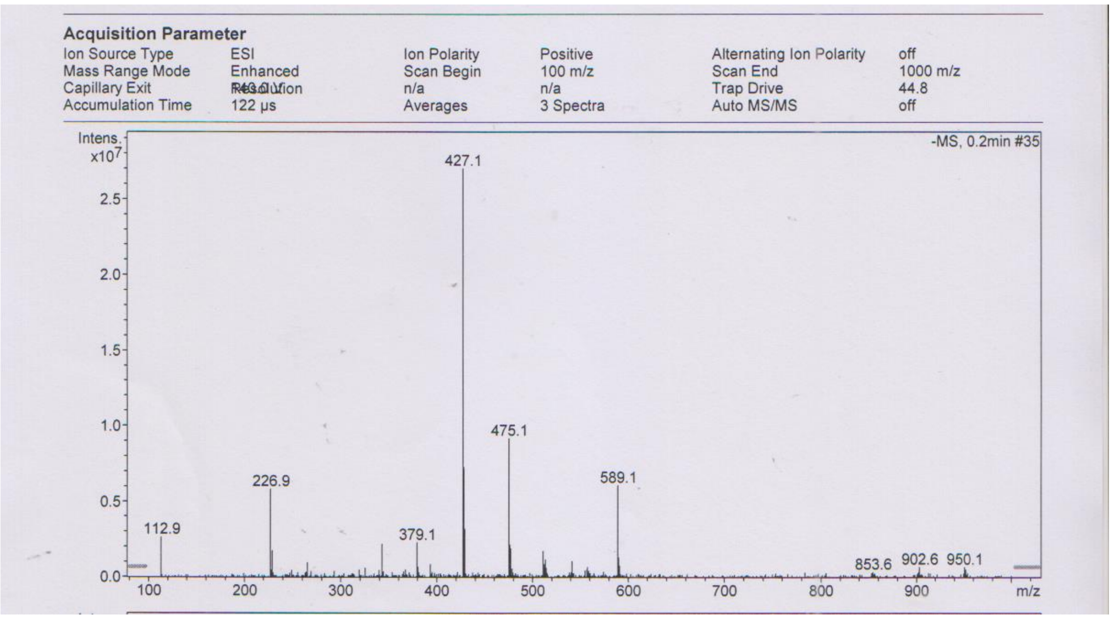

Figure S10. ESIMS spectrum of compound **1**

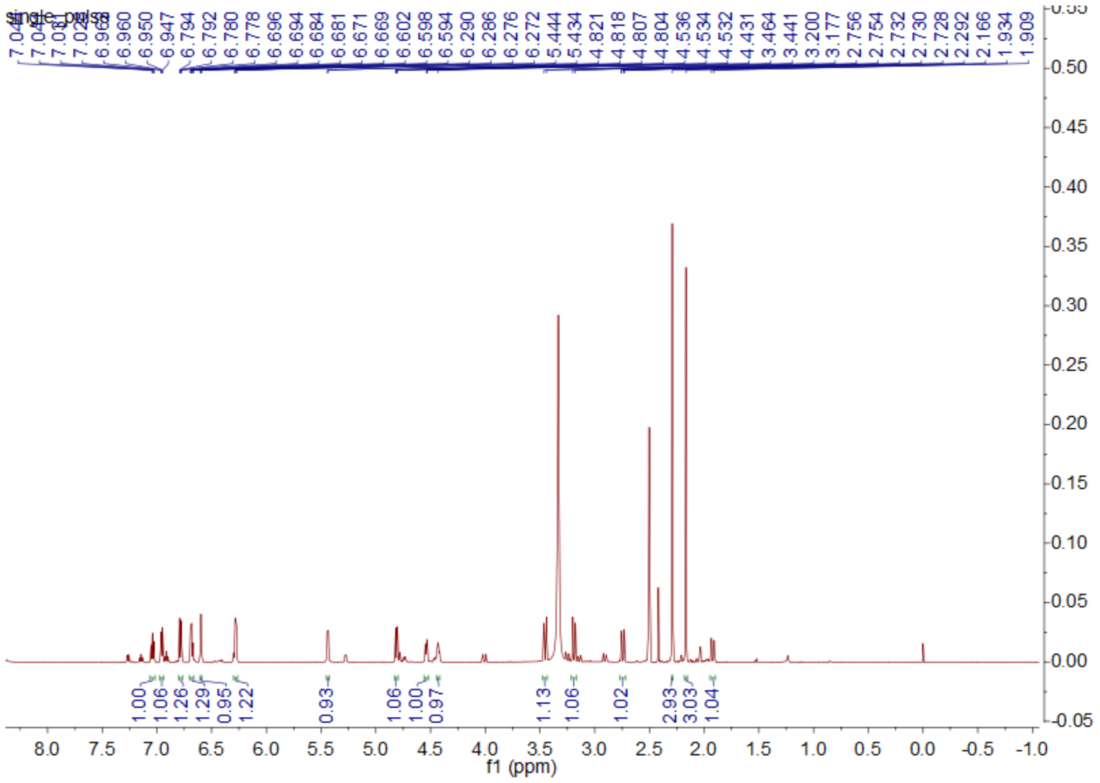

Figure S11. ^{1}H NMR (500 MHz, DMSO-d_6) spectrum of compound **2**

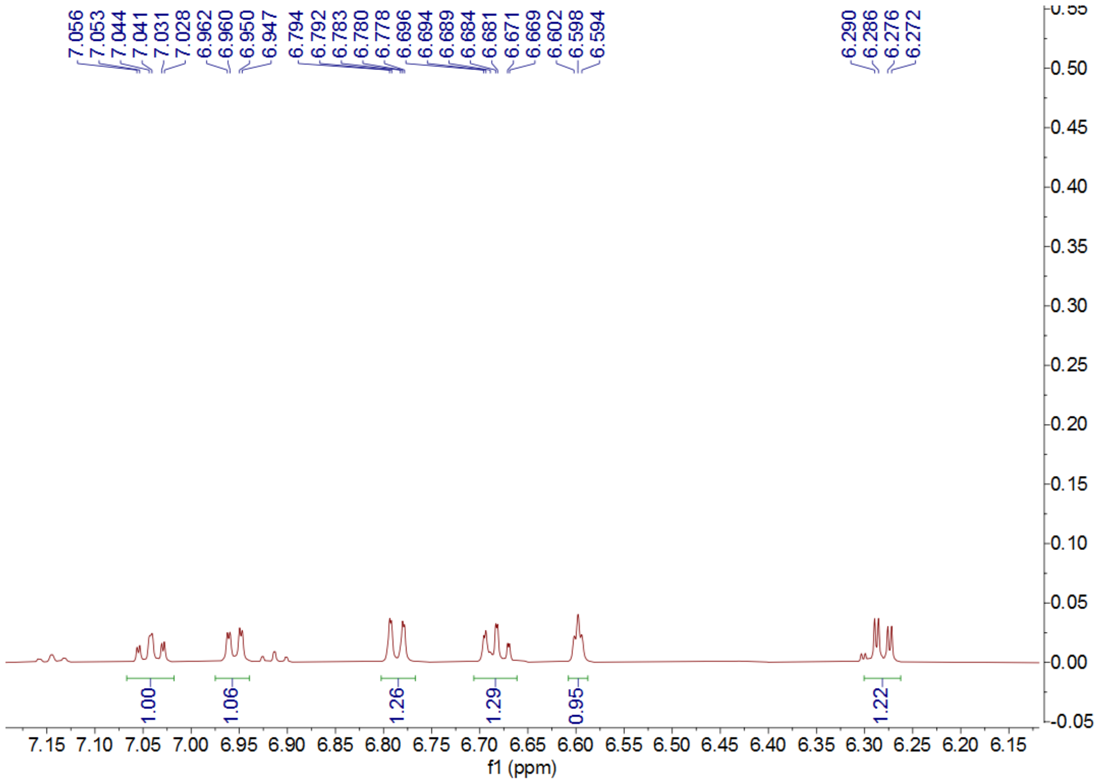

Figure S12. Partial ^{1}H NMR (500 MHz, DMSO-d_6) spectrum of compound **2**

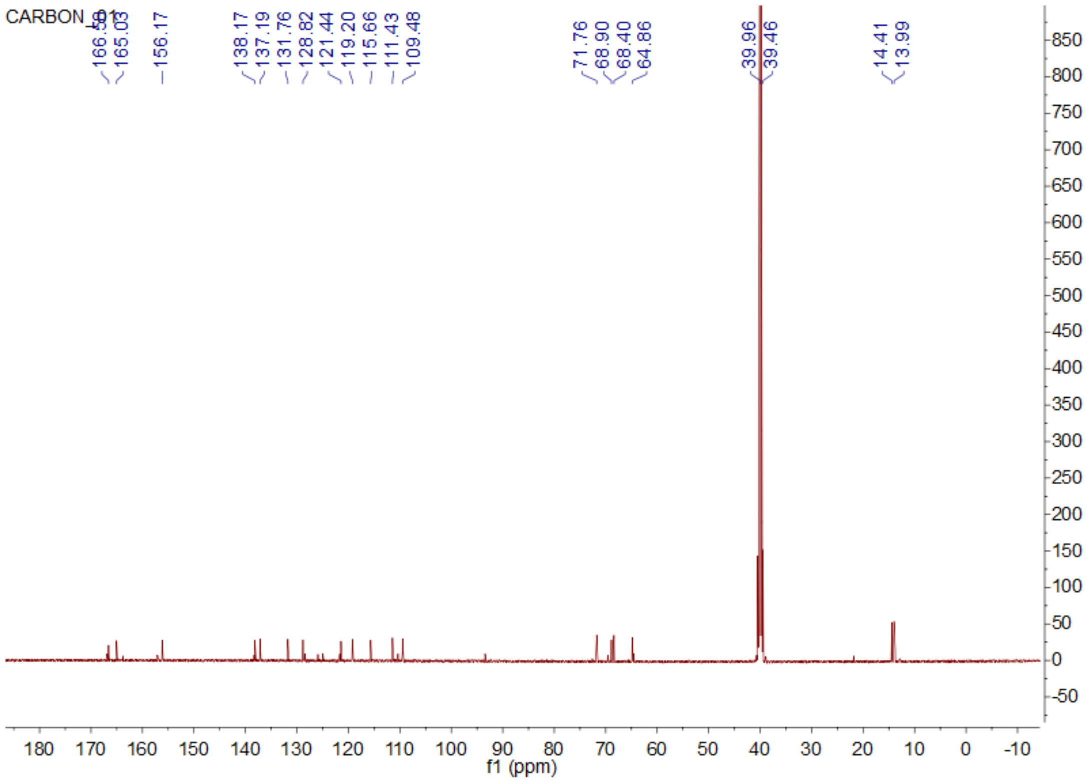

Figure S13. ^{13}C NMR (125 MHz, DMSO-d_6) spectrum of compound **2**

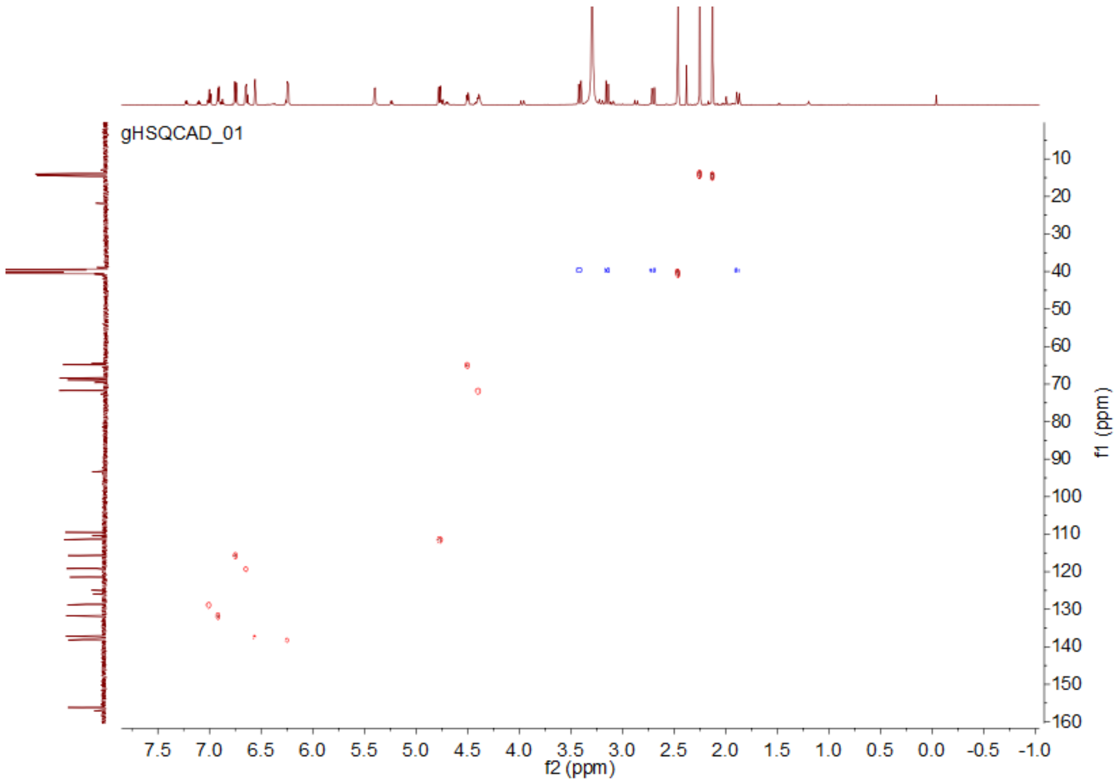

Figure S14. HSQC (DMSO-d_6) spectrum of compound **2**

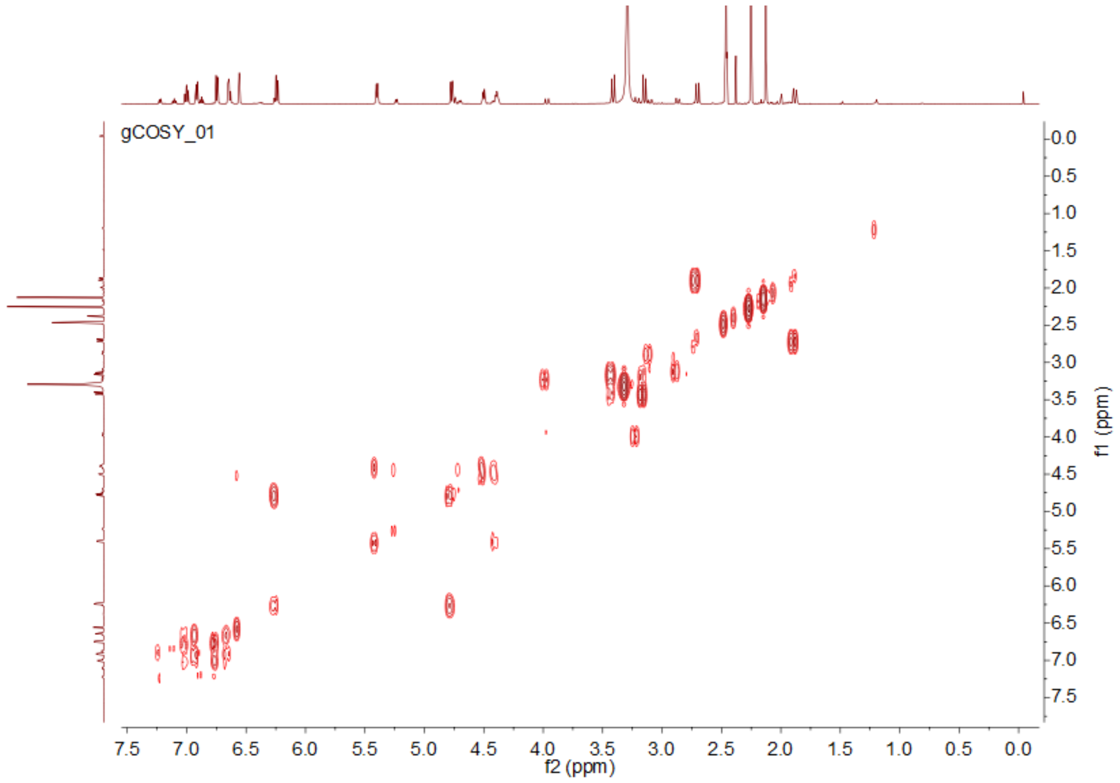

Figure S15. ^{1}H–^{1}H COSY (DMSO-d_6) spectrum of compound **2**

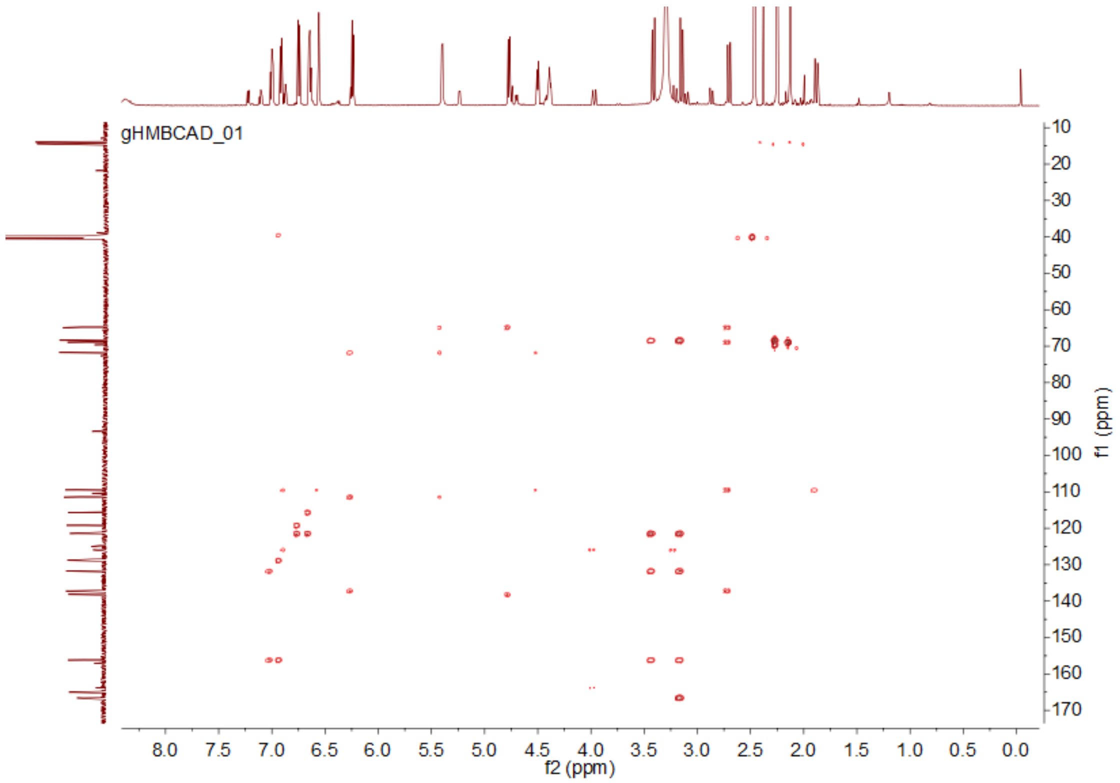

Figure S16. HMBC (DMSO-d_6) spectrum of compound **2**

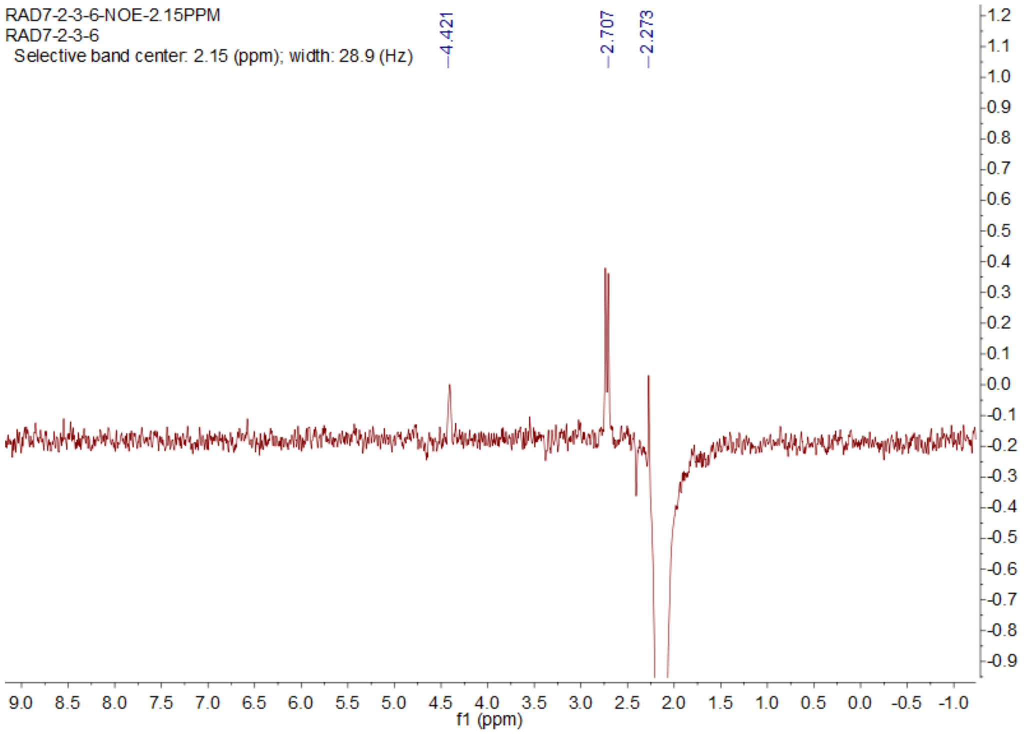

Figure S17. NOE (DMSO-d_6) spectrum of compound **2**

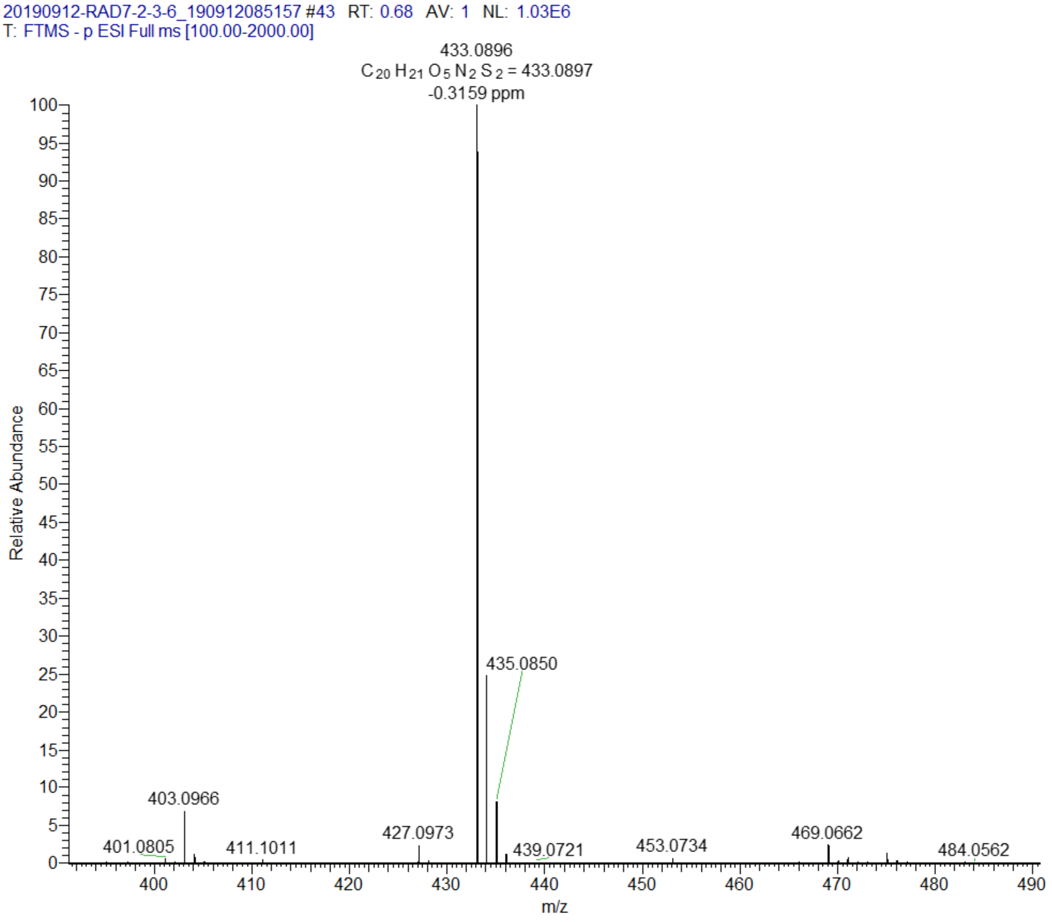

Figure S18. HRESIMS spectrum of compound **2**

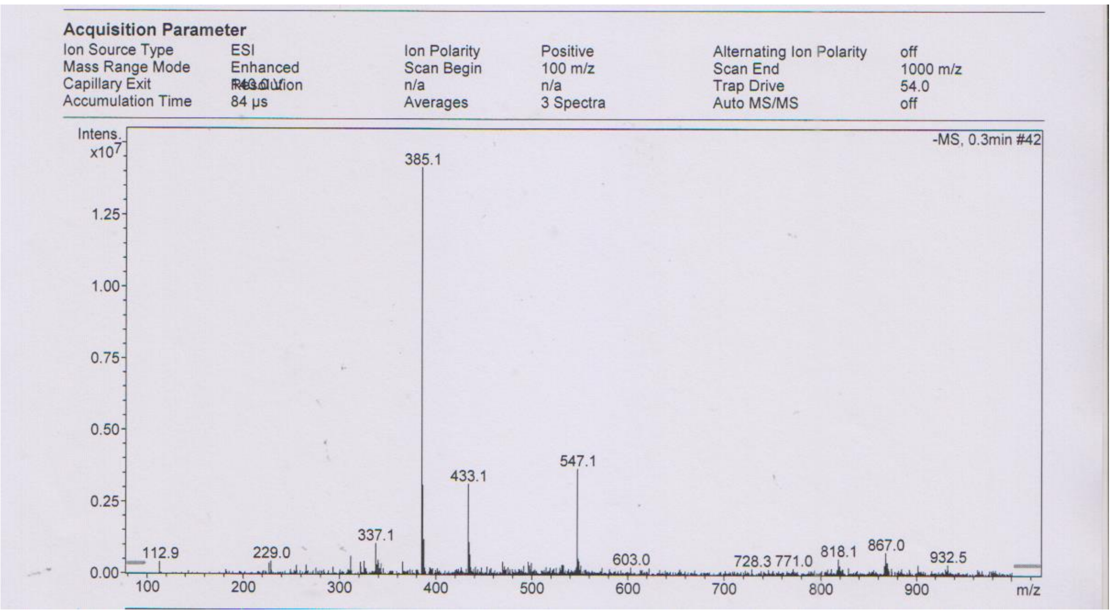

Figure S19. ESIMS spectrum of compound **2**

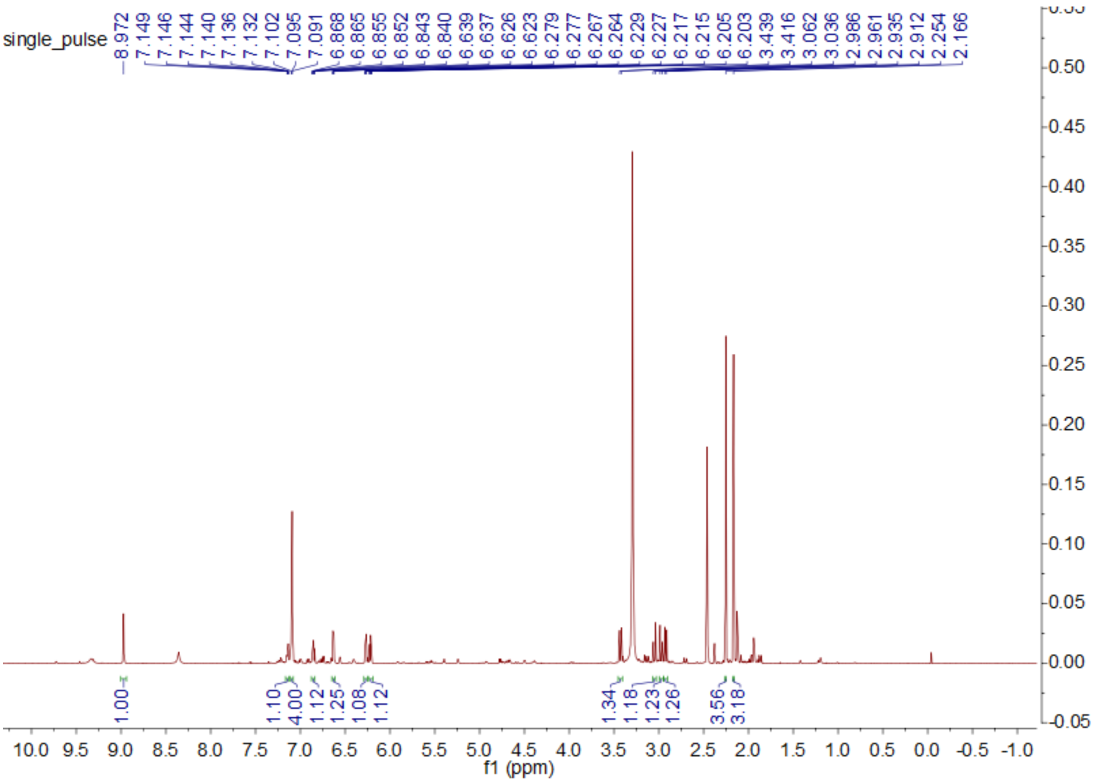

Figure S20. ^{1}H NMR (500 MHz, DMSO-d_6) spectrum of compound **3**

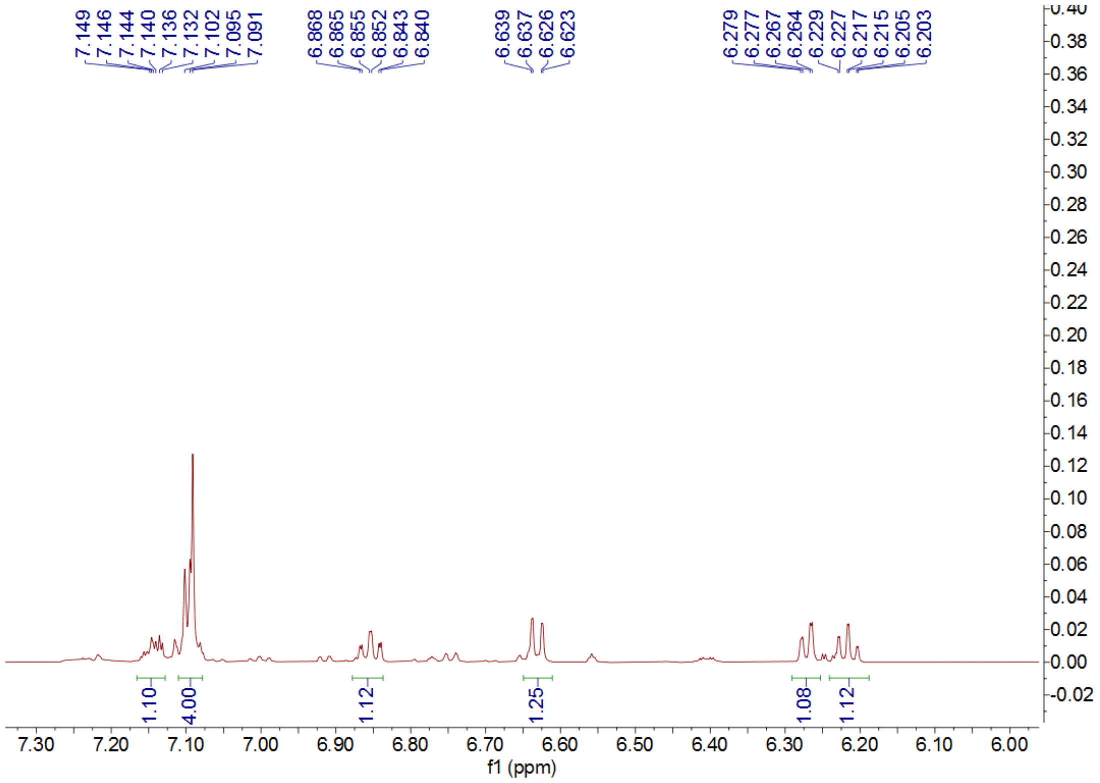

Figure S21. Partial ^{1}H NMR (500 MHz, DMSO-d_6) spectrum of compound **3**

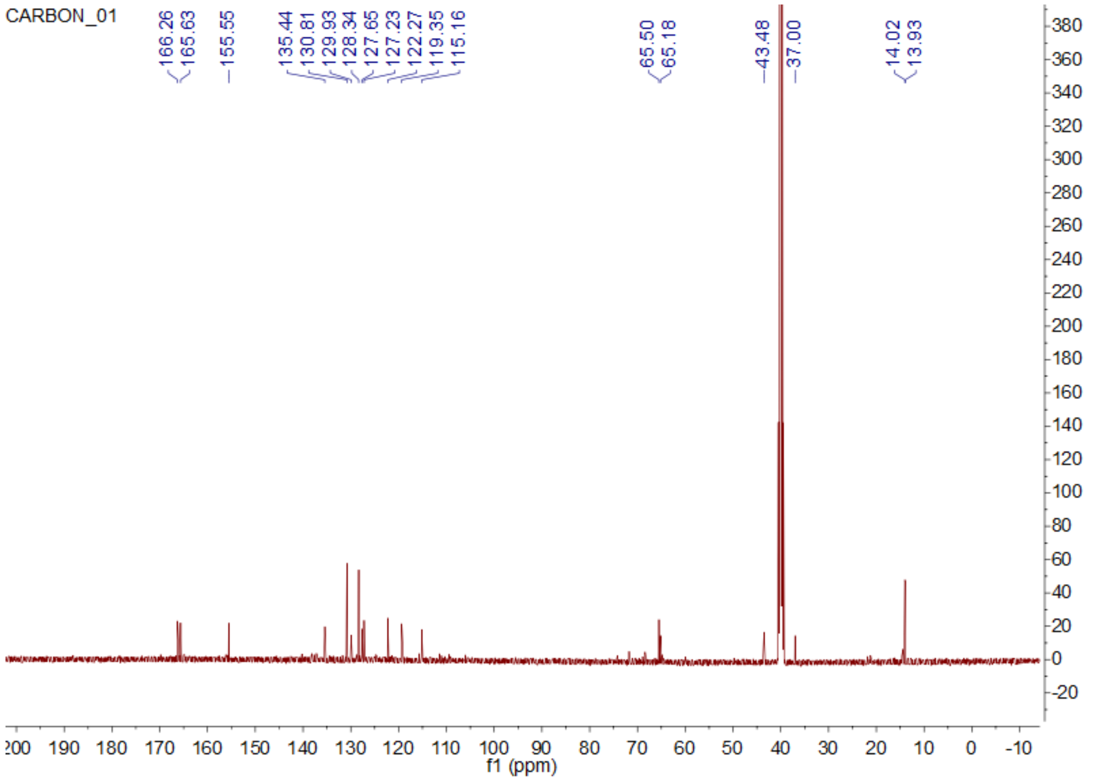

Figure S22. ^{13}C NMR (125 MHz, DMSO-d_6) spectrum of compound **3**

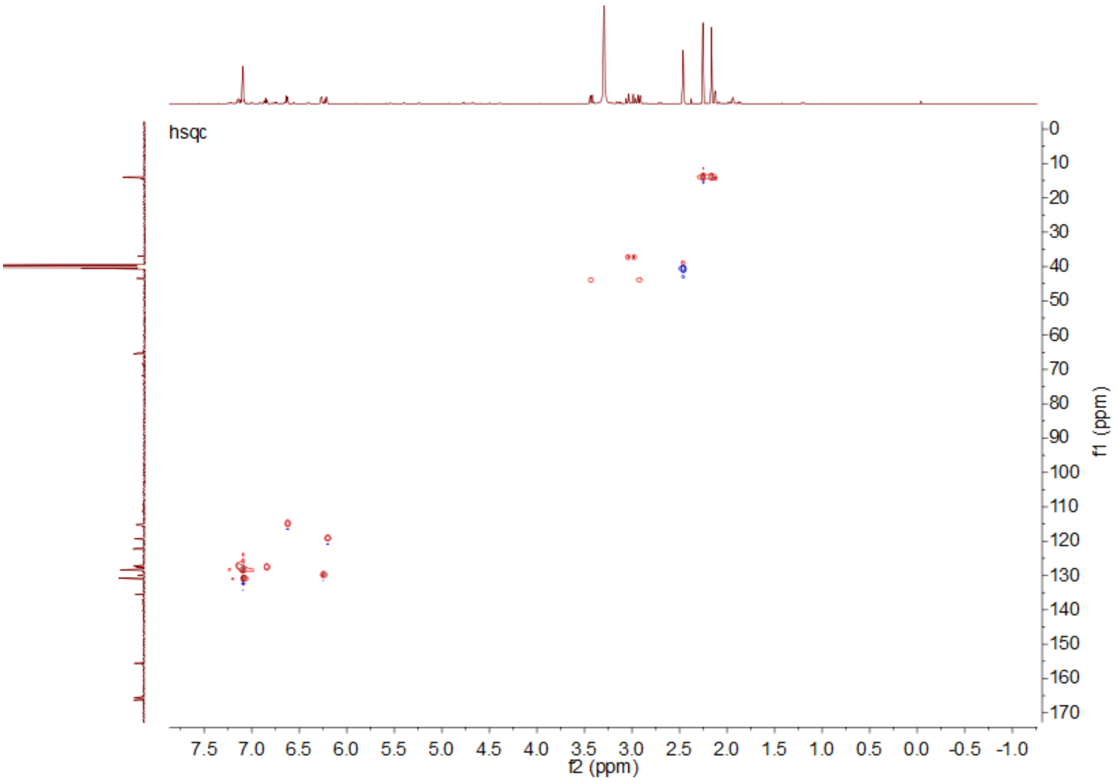

Figure S23. HSQC (DMSO-d_6) spectrum of compound **3**

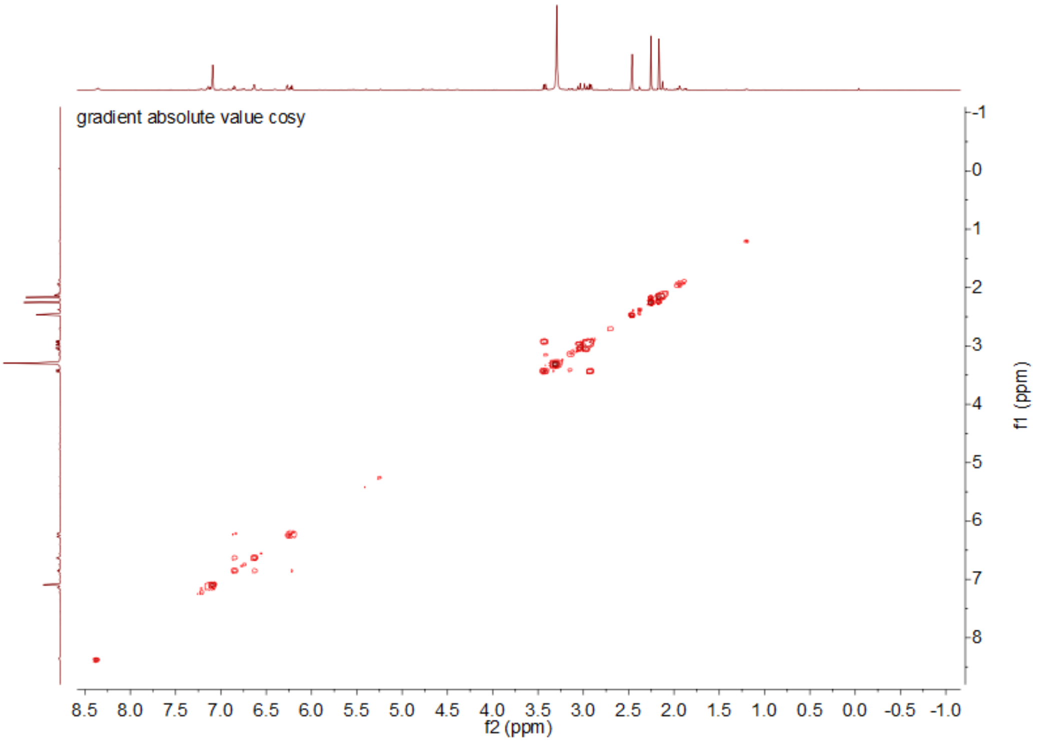

Figure S24. ^{1}H–^{1}H COSY (DMSO-d_6) spectrum of compound **3**

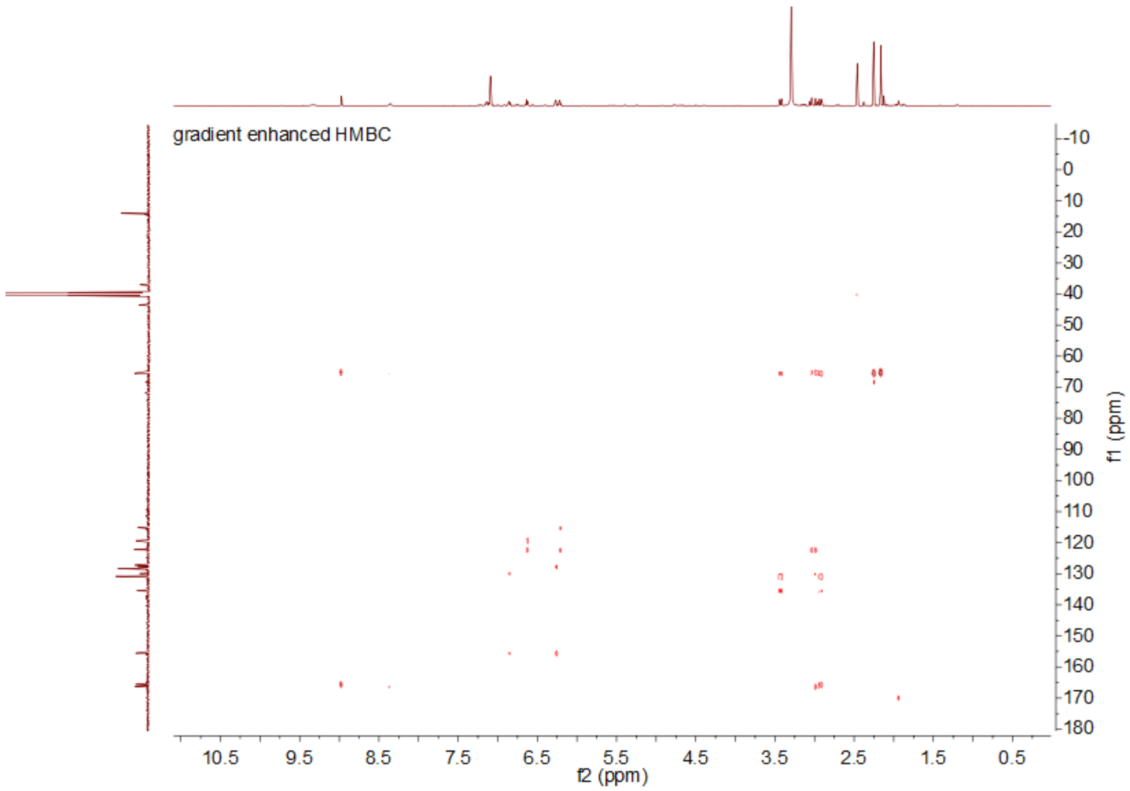

Figure S25. HMBC (DMSO-d_6) spectrum of compound **3**

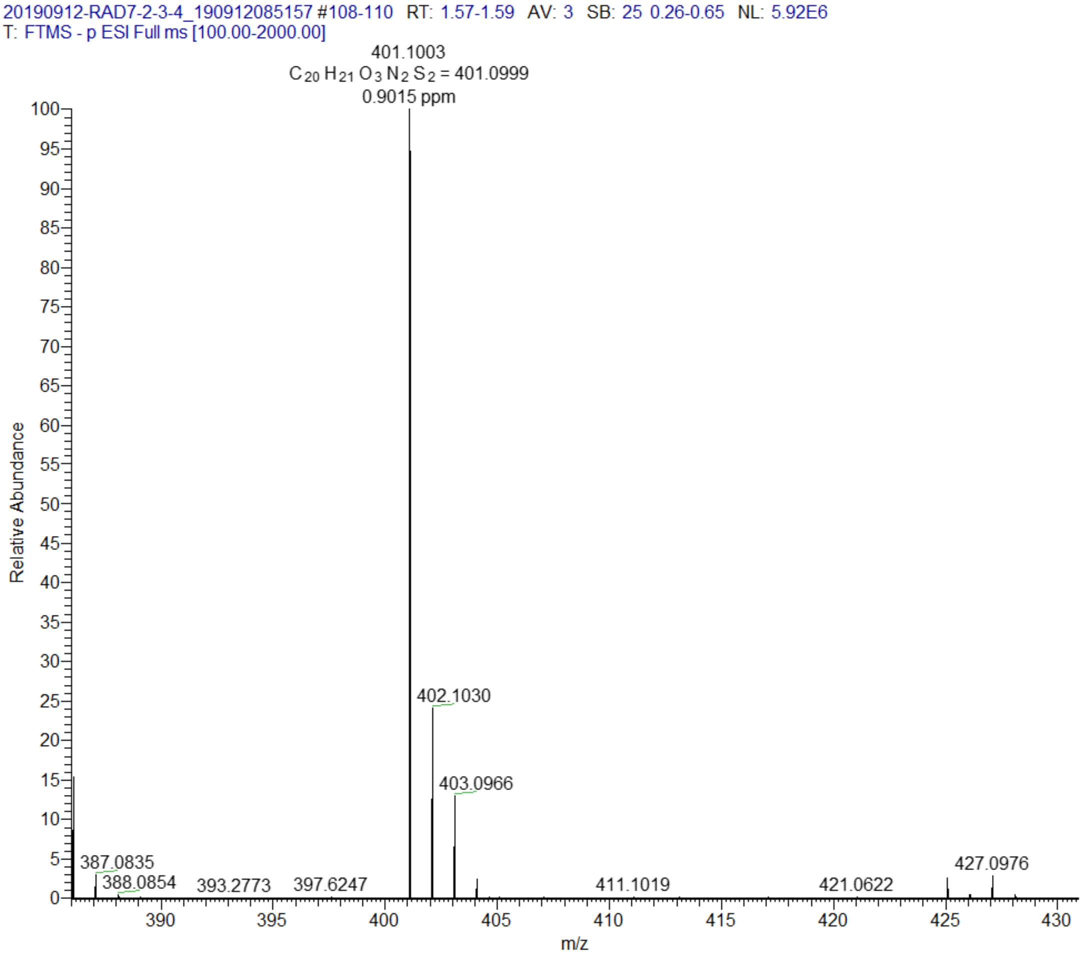

Figure S26. HRESIMS spectrum of compound **3**

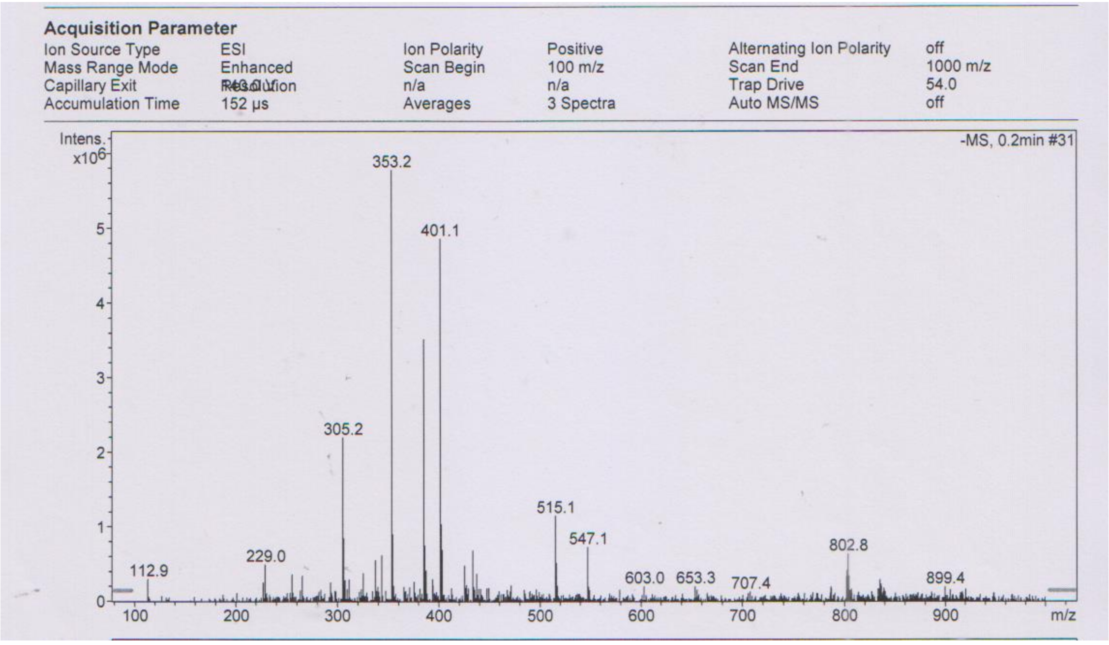

Figure S27. ESIMS spectrum of compound **3**

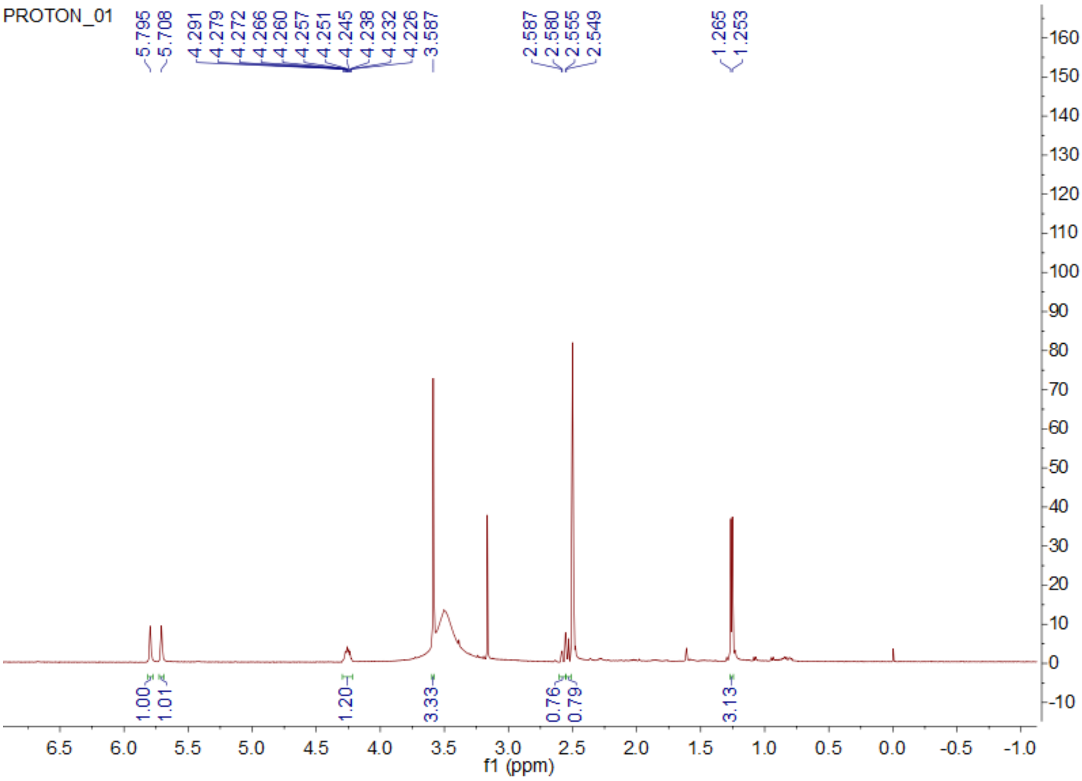

Figure S28. ^{1}H NMR (500 MHz, DMSO-d_6) spectrum of compound **8**

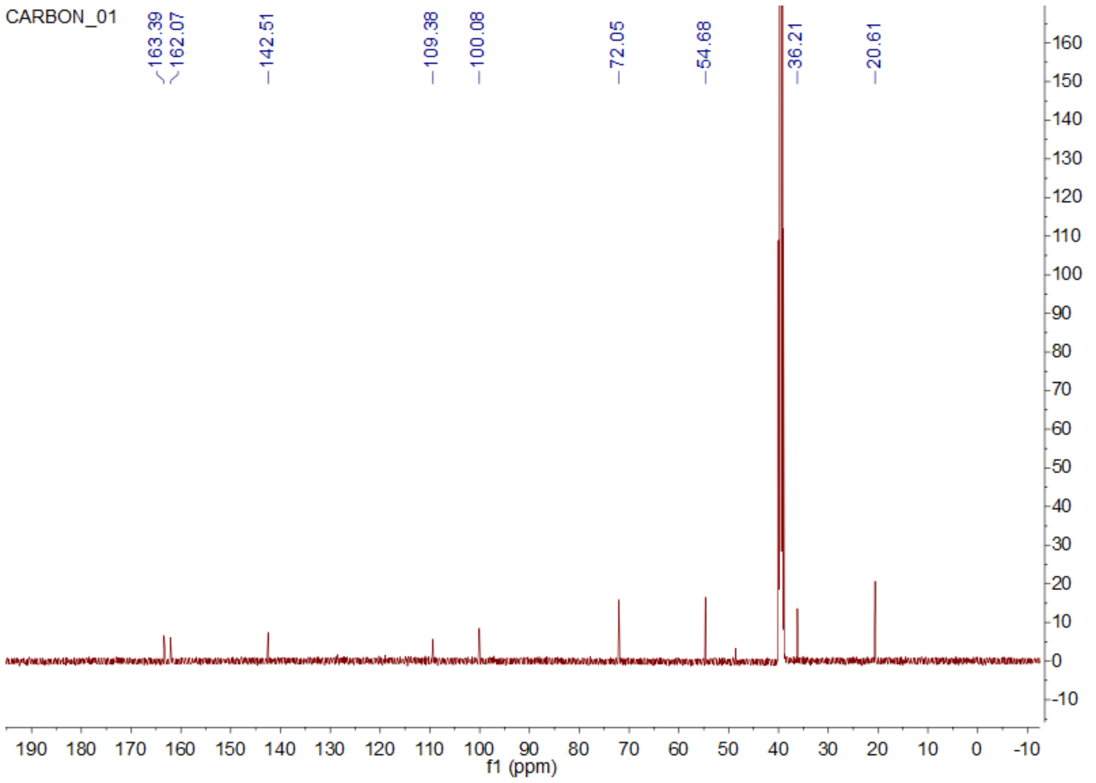

Figure S29. ^{13}C NMR (125MHz, DMSO-d_6) spectrum of compound **8**

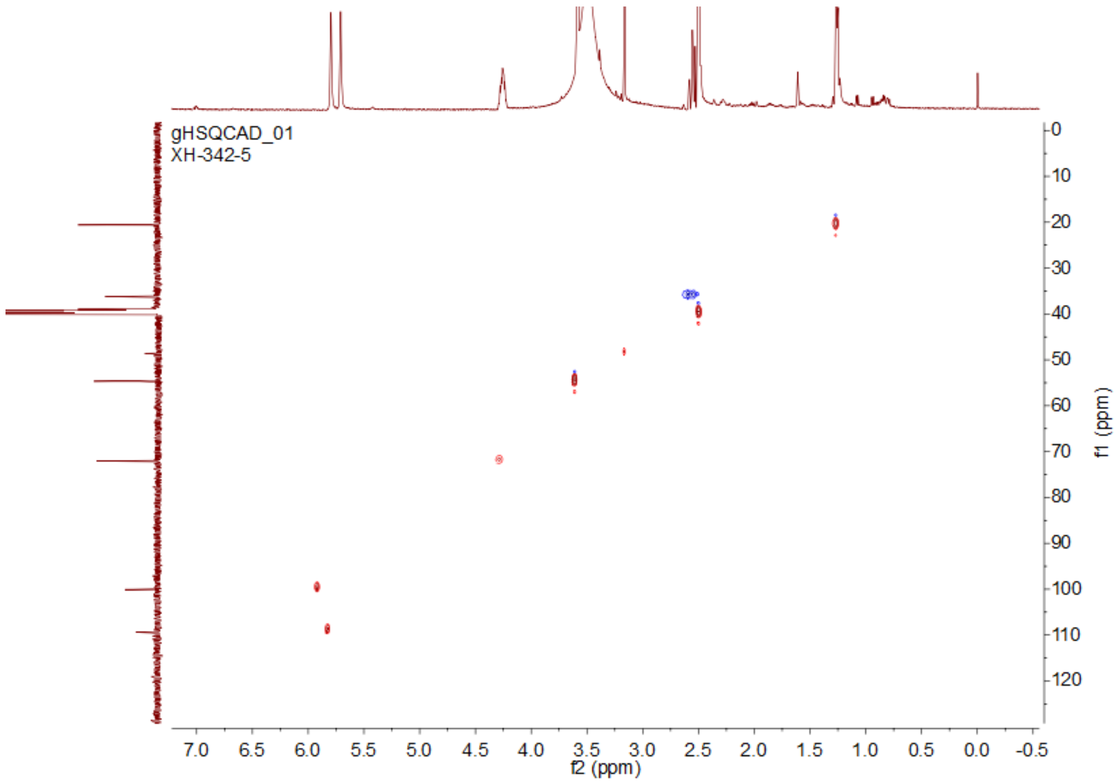

Figure S30. HSQC (DMSO-d_6) spectrum of compound **8**

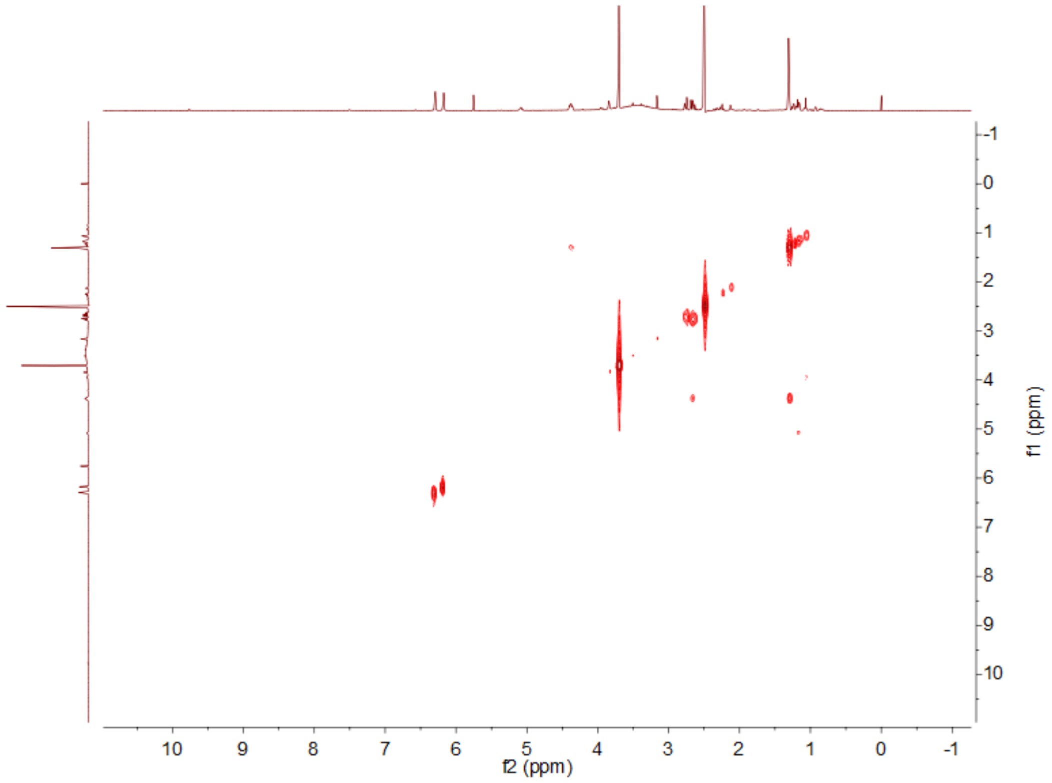

Figure S31. ^{1}H–^{1}H COSY (DMSO-d_6) spectrum of compound **8**

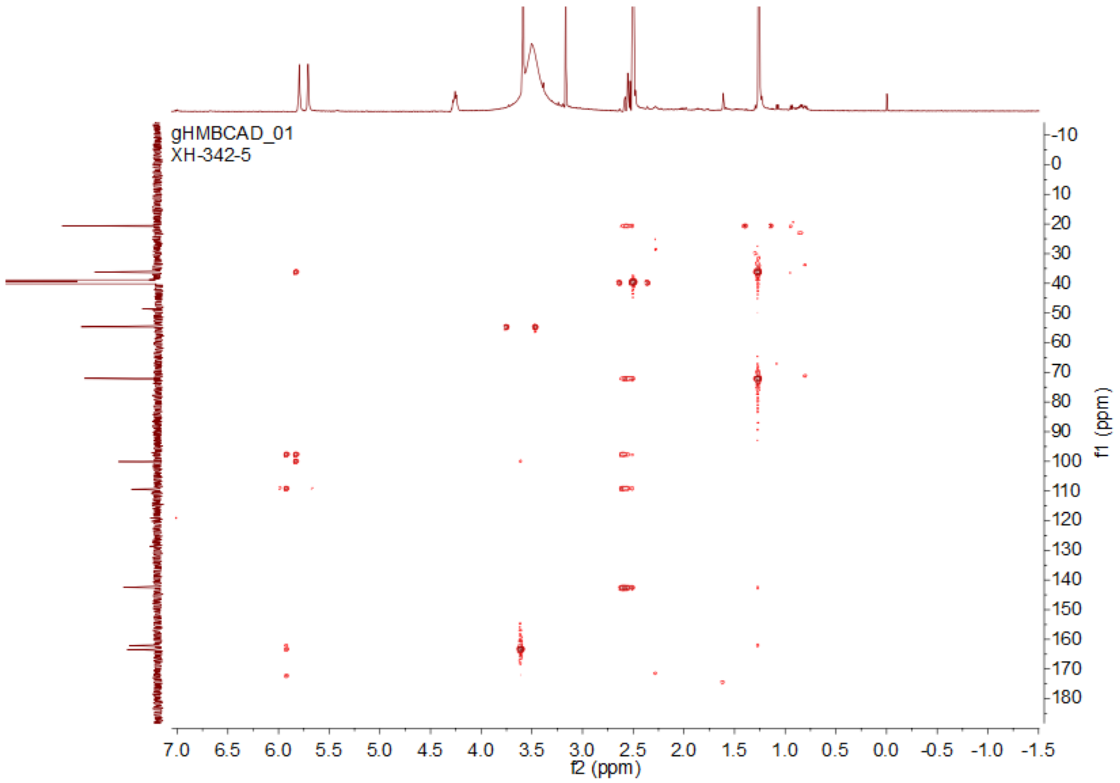

Figure S32. HMBC (DMSO-d_6) spectrum of compound **8**

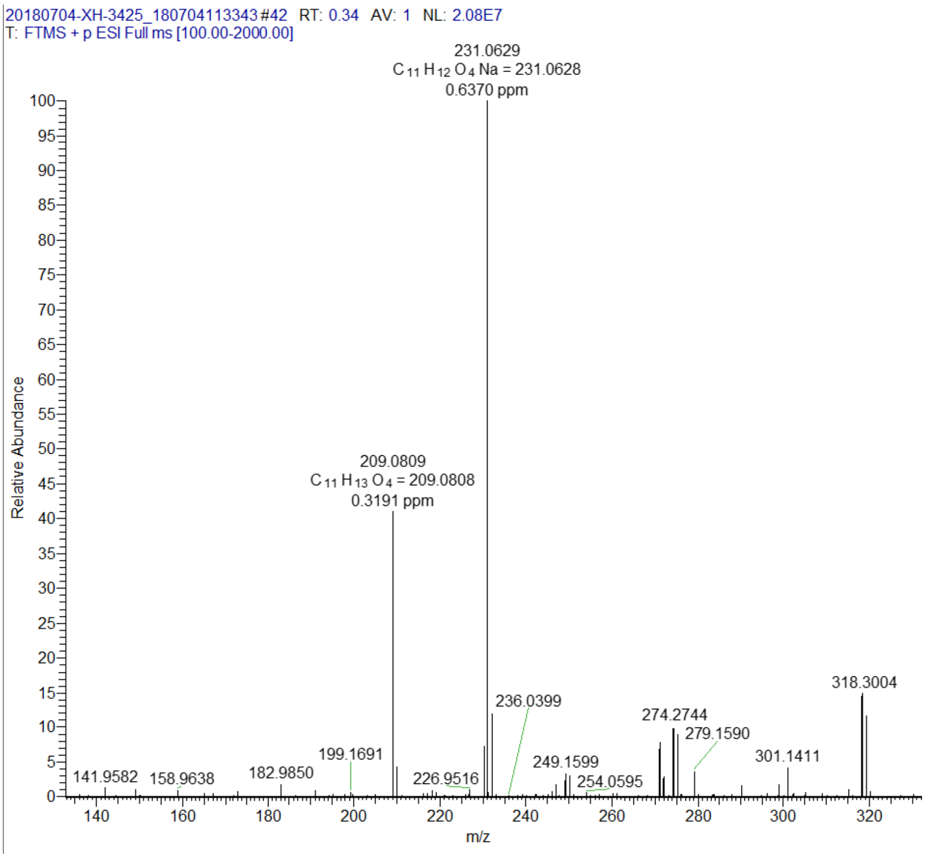

Figure S33. HRESIMS spectrum of compound **8**

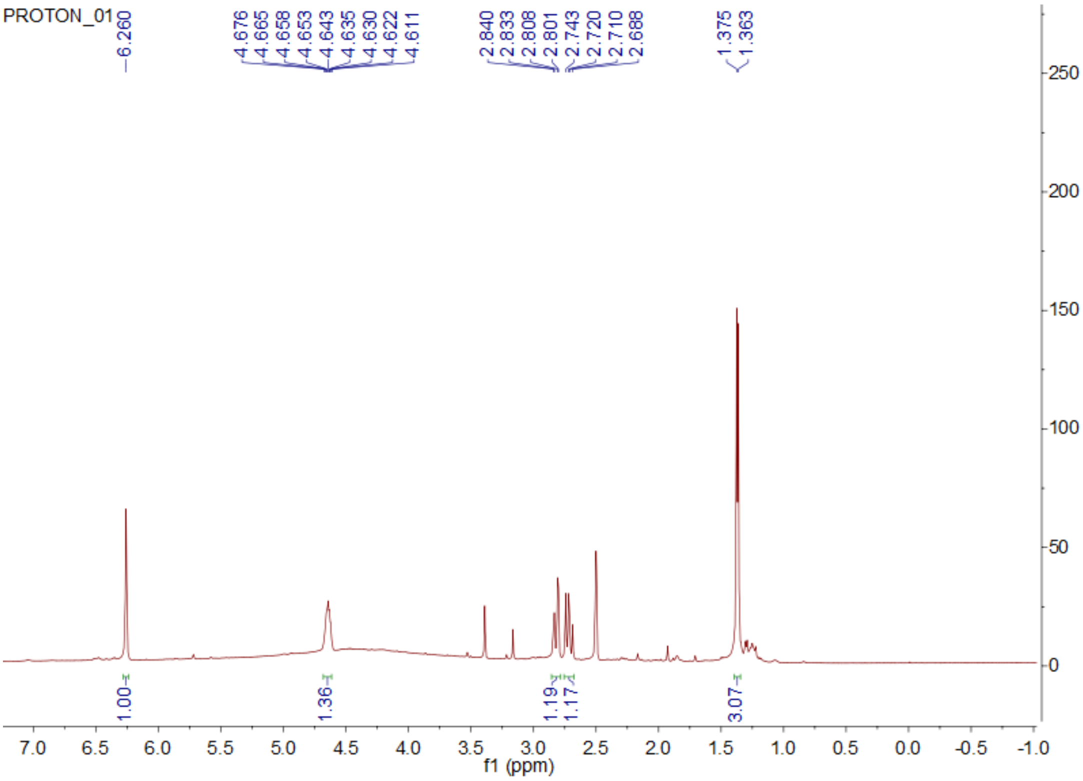

Figure S34. ^{1}H NMR (500 MHz, DMSO-d_6) spectrum of compound **9**

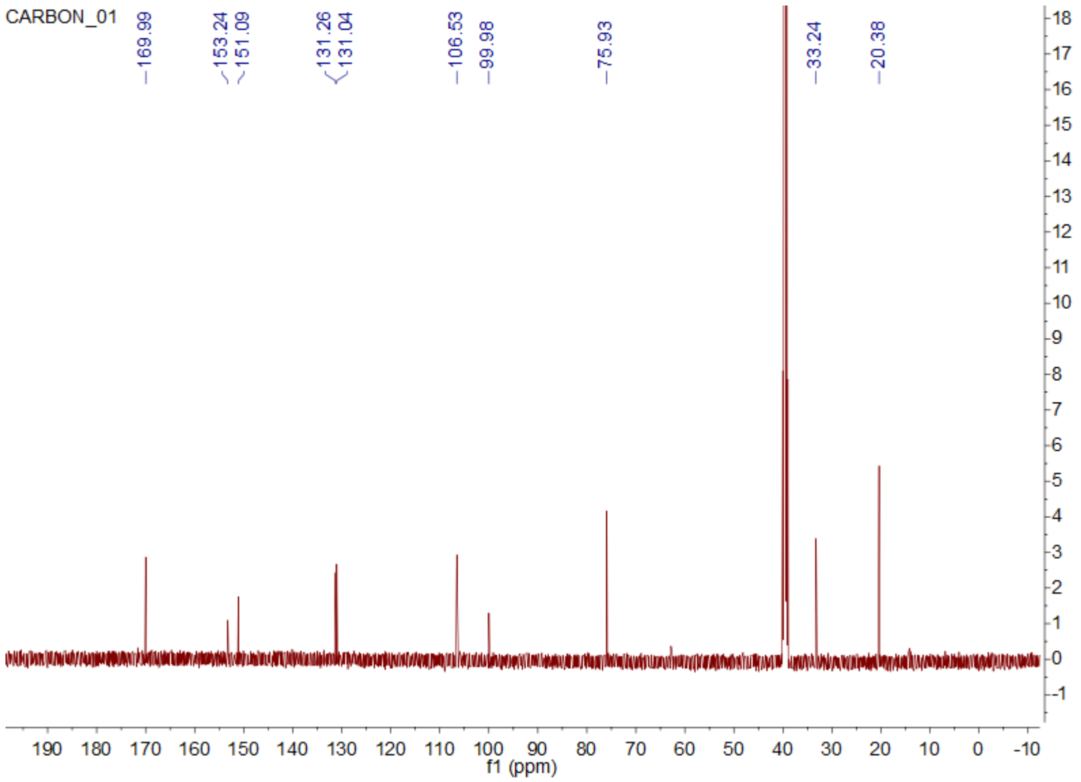

Figure S35. ^{13}C NMR (125MHz, DMSO-d_6) spectrum of compound **9**

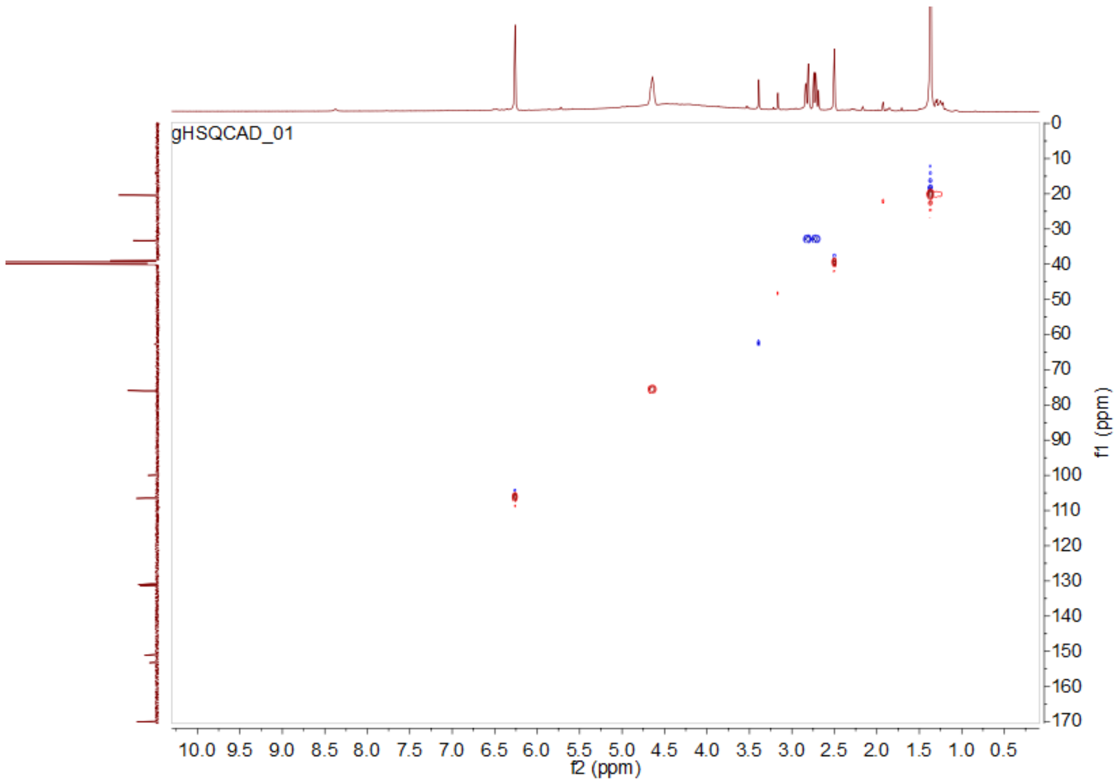

Figure S36. HSQC (DMSO-d_6) spectrum of compound **9**

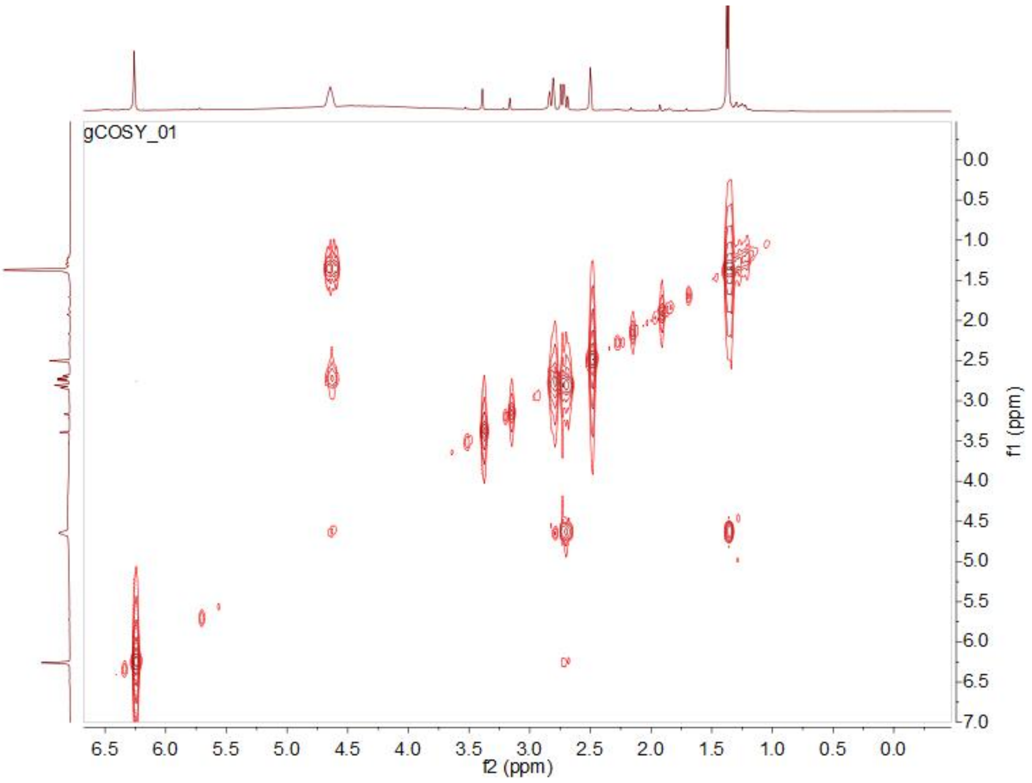

Figure S37. ^{1}H–^{1}H COSY (DMSO-d_6) spectrum of compound **9**

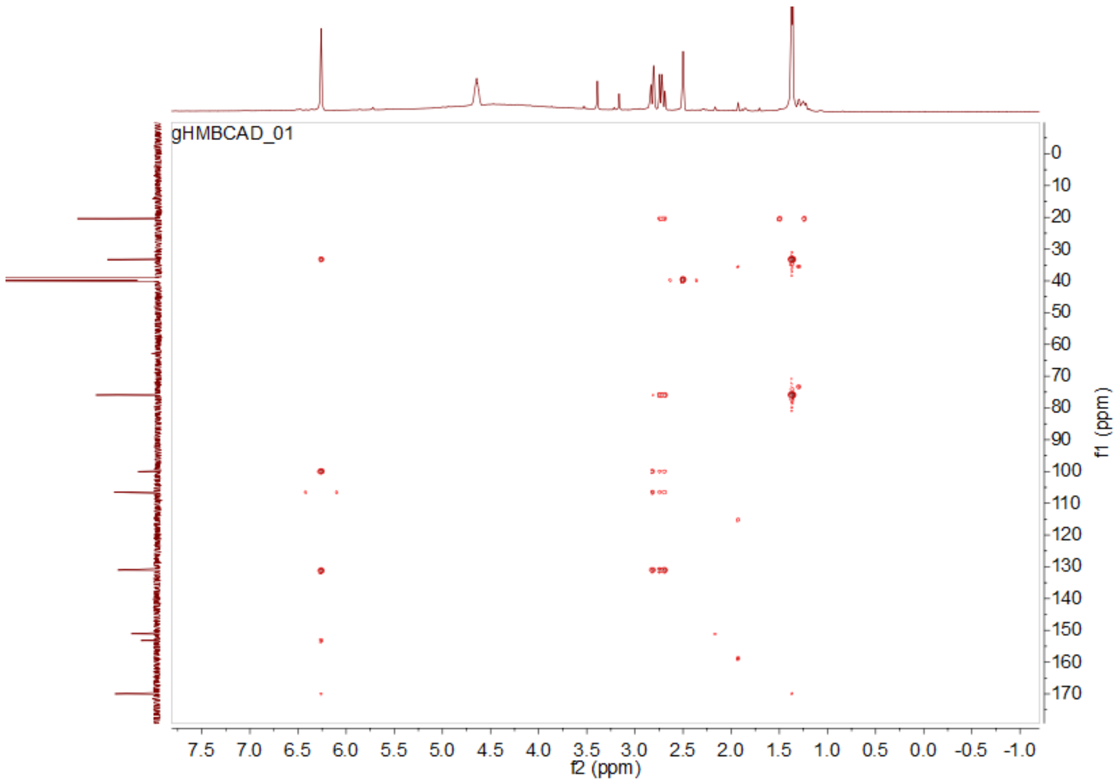

Figure S38. HMBC (DMSO-d_6) spectrum of compound **9**

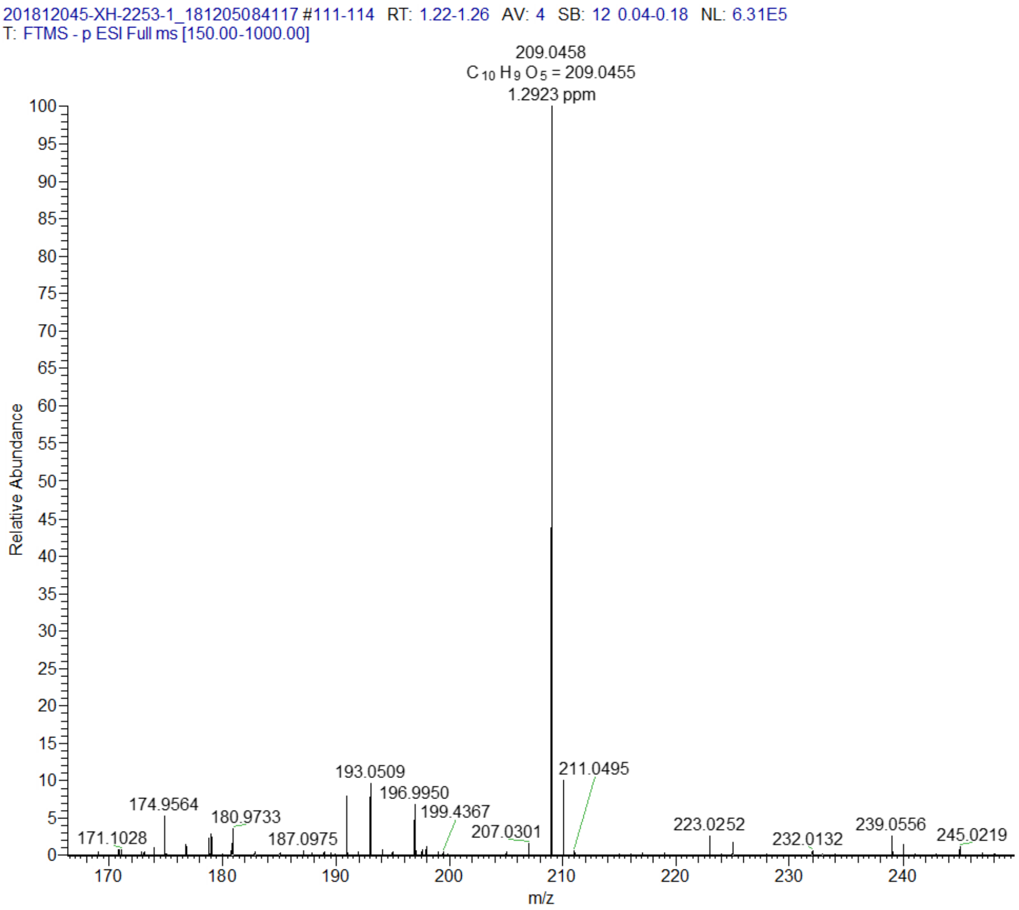

Figure S39. HRESIMS spectrum of compound **9**

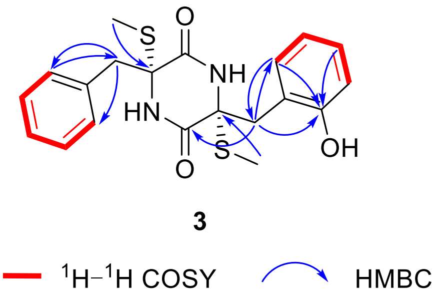

Figure S40. Key HMBC correlations of compound **3**

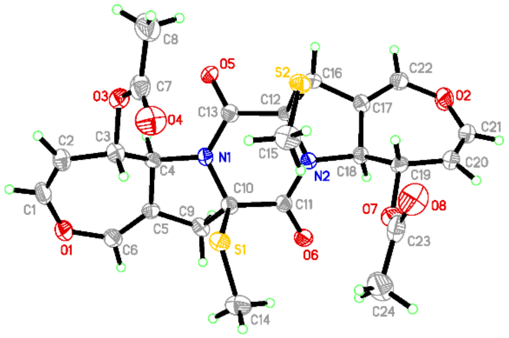

Figure S41. Crystal diagram of compound **6** (CCDC:1911625)

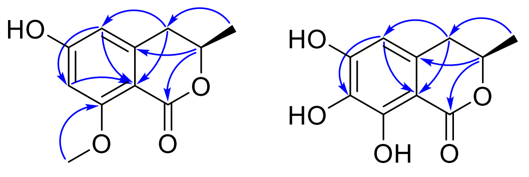

8 **9**

Key HMBC correlation

Figure S42. Key HMBC correlations of compounds **8** and **9**

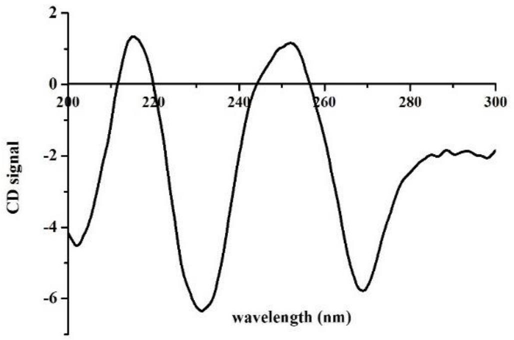

Figure S43. The CD spectrum of compound **8**

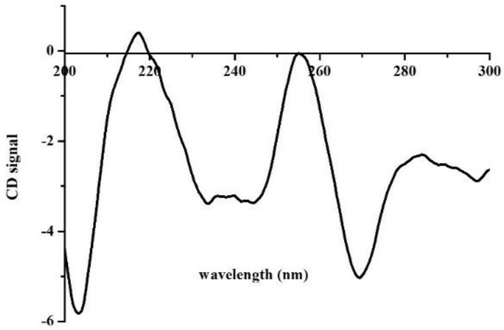

Figure S44. The CD spectrum of compound **9**
